# Incorporating Concomitant Medications into Genome-Wide Analyses for the Study of Complex Disease and Drug Response

**DOI:** 10.3389/fgene.2016.00138

**Published:** 2016-08-17

**Authors:** Hillary T. Graham, Daniel M. Rotroff, Skylar W. Marvel, John B. Buse, Tammy M. Havener, Alyson G. Wilson, Michael J. Wagner, Alison A. Motsinger-Reif, the ACCORD/ACCORDion Investigators, W.T. Friedewald

**Affiliations:** Author Affiliations: Steering Committee: Canadian CCN: Population Health Research Institute, Hamilton General Hospital, Canadian Diabetes Outcome Researchers (CANDOR Network), Hamilton, Ontario, Canada; Canadian clinical sites: McMaster Medical Centre, Hamilton, Ontario, Canada; Six Nations Health Services, Ohsweken, Ontario, Canada; Diabetes, Hypertension and Cholesterol Centre, University of Calgary, Calgary, Alberta, Canada; Memorial University of Newfoundland, St. John's, Newfoundland, Canada; University of Alberta, Edmonton, Alberta, Canada; Centre de Recherche Clinique de Laval, Laval, Quebec, Canada; St. Joseph's Health Care London, London, Ontario, Canada; Ottawa Hospital Research Institute, Division of Endocrinology and Metabolism, Ottawa, Ontario, Canada; Royal Victoria Hospital, Montreal, Quebec, Canada; St. Michael's Hospital, Toronto, Ontario, Canada; Vancouver General Hospital, Vancouver, British Columbia, Canada; Health Sciences Centre Diabetes Research Group, Winnipeg, Manitoba, Canada; Nova Scotia Health Authority, Queen Elizabeth II Health Sciences Centre, Halifax, Nova Scotia, Canada; Western CCN: University of Washington, Seattle, WA; Western clinical sites: Northridge Hospital Medical Center, Cardiovascular Center, Northridge, CA; White Memorial Medical Center, Clinical Hypertension Services, Los Angeles, CA; University of Washington Medical Center at Roosevelt, Family Medical Center, Seattle, WA; Idaho State University, Department of Family Medicine, Pocatello, ID; Naval Medical Center San Diego, Cardiology Division, San Diego, CA; Oregon Health & Science University, Section of Diabetes, Portland, OR; Washington State University, Spokane, WA; Kaiser Endocrine Clinic, San Diego, CA; Whittier Institute for Diabetes, Clinical Trials Department, La Jolla, CA; Minnesota-Iowa CCN: Berman Center for Outcomes & Clinical Research, Minneapolis, MN; Health Partners Research Foundation, Minneapolis, MN; Minnesota-Iowa clinical sites: Hennepin ACCORD Clinic, Minneapolis, MN; International Diabetes Center, Minneapolis, MN; University of Minnesota, Minneapolis, MN; University of Minnesota, Phalen Village Clinic, St. Paul, MN; Riverside Health Partners Clinic, Department of Endocrinology, Minneapolis, MN; University of Iowa, Health Care Diabetes Clinical Research and Programs, Iowa City, IA; Ohio-Michigan CCN: Case Western Reserve University, Division of Clinical and Molecular Endocrinology, Cleveland, OH; Ohio-Michigan clinical sites: University Hospitals of Cleveland, Division of Endocrinology, and University Hospitals Westlake Medical, Cleveland, OH; St. Vincent Charity Hospital, Lipid Research Center, Cleveland, OH; University Suburban Health Center, South Euclid, OH; Cleveland Veterans Affairs (VA) Medical Center (VAMC), Department of Medicine, and Ravenna Community Based Outpatient Clinic, Cleveland, OH; The Cleveland Clinic Foundation, Cleveland, OH; Your Diabetes Endocrine Nutrition Group, Mentor, OH; Medical University of Ohio, Department of Medicine, Ruppert Health Center, Toledo, OH; The Ohio State University Medical Center, Division of Endocrinology, Diabetes and Metabolism, Columbus, OH; University of Cincinnati/VA Medical Center, Research Service, Cincinnati, OH; Henry Ford Health System-New Center One, Detroit, MI; Grunberger Diabetes Institute, Bloomfield Hills, MI; Northeastern CCN: Columbia University College of Physicians and Surgeons, New York, NY; Northeastern clinical sites: Jacobi Medical Center, Bronx, NY; Albert Einstein General Clinical Research Center, Bronx, NY; Cornell Internal Medicine Associates, New York, NY; The Diabetes Care and Information Center of New York, Flushing, NY; The Cooper Health System, Cherry Hill, NJ; The Cooper Health System, Cherry Hill, NJ; Great Lakes Medical Clinic Research, Westfield, NY; Naomi Berrie Diabetes Center, New York, NY; Ambulatory Care Network at Columbia University, New York, NY; Irving Diabetes Research Unit, New York, NY; State University of New York Downstate Medical Center, Brooklyn, NY; Kings County, Brooklyn, NY; The Cooper Health System, Cherry Hill, NJ; Southeastern CCN: Wake Forest University School of Medicine, Department of Public Health Sciences, Winston-Salem, NC; Southeastern clinical sites: Duke University Medical Center, Durham, NC; Constant Care, Inc., Valdosta, GA; Wake Forest University School of Medicine, Department of Geriatrics/Gerontology, Winston-Salem, NC; Downtown Health Plaza, Winston-Salem, NC; University of North Carolina, Diabetes Care Center, Chapel Hill, NC; Holston Medical Group, Kingsport, TN; Carolinas Medical Center Family Practice, Charlotte, NC; Robeson Health Care Corporation, Fairmont Clinic, Fairmont, NC; Robeson Health Care Corporation, Julian T. Pierce Clinic, Pembroke, NC; Wake Forest University School of Medicine, Departments of Internal Medicine and Endocrinology, Winston-Salem, NC; Tulane University Health Science Center, New Orleans, LA; Kaiser Permanente, Clinic Atlanta Crescent Medical Center, Tucker, GA; VA CCN: Memphis VAMC, Memphis, TN; VA clinical sites: Memphis VAMC, Hypertension/Lipid Research Clinic, Memphis, TN; Baltimore VAMC, Baltimore, MD; Carl T. Hayden VAMC, Phoenix, AZ; Atlanta VAMC Medical Service, Decatur, GA; Ralph H. Johnson VAMC, Primary Care, Charleston, SC; G. V. (Sonny) Montgomery VAMC, Research Department, Jackson, MS; VA NY Harbor Healthcare System, New York, NY; Washington VAMC, Washington, DC; St. Louis VAMC, St. Louis, MO; Central Arkansas Veterans Healthcare System,John L. McClellan Memorial Veterans Hospital, Little Rock, AR; Coordinating Center: Wake Forest University School of Medicine, Winston-Salem, NC; Drug Distribution Center: VA Cooperative Studies Program Clinical Research Pharmacy Coordinating Center, Albuquerque, NM; ECG Reading Center: Wake Forest University School of Medicine, Winston-Salem, NC; Central Chemistry Laboratory: Northwest Lipid Research Laboratories, Seattle, WA; ACCORD-MIND MRI Reading Center: University of Pennsylvania, Philadelphia, PA; Fundus Photograph Reading Center: University of Wisconsin Medical School, Madison, WI; Project Office: National Heart, Lung, and Blood Institute (NHLBI), Bethesda, MD; National Institute of Diabetes and Digestive and Kidney Diseases (NIDDK), Bethesda, MD; National Institute on Aging (NIA), Bethesda, MD; National Eye Institute (NEI), Bethesda, MD; Centers for Disease Control and Prevention (CDC), Atlanta, GA; ^1^Department of Statistics, North Carolina State UniversityRaleigh, NC, USA; ^2^Bioinformatics Research Center, North Carolina State UniversityRaleigh, NC, USA; ^3^Department of Medicine, University of North Carolina School of MedicineChapel Hill, NC, USA; ^4^Center for Pharmacogenomics and Individualized Therapy, University of North Carolina at Chapel HillChapel Hill, NC, USA

**Keywords:** clinical trial, genomics, drug response, precision medicine, bioinformatics

## Abstract

Given the high costs of conducting a drug-response trial, researchers are now aiming to use retrospective analyses to conduct genome-wide association studies (GWAS) to identify underlying genetic contributions to drug-response variation. To prevent confounding results from a GWAS to investigate drug response, it is necessary to account for concomitant medications, defined as any medication taken concurrently with the primary medication being investigated. We use data from the Action to Control Cardiovascular Disease (ACCORD) trial in order to implement a novel scoring procedure for incorporating concomitant medication information into a linear regression model in preparation for GWAS. In order to accomplish this, two primary medications were selected: thiazolidinediones and metformin because of the wide-spread use of these medications and large sample sizes available within the ACCORD trial. A third medication, fenofibrate, along with a known confounding medication, statin, were chosen as a proof-of-principle for the scoring procedure. Previous studies have identified SNP rs7412 as being associated with statin response. Here we hypothesize that including the score for statin as a covariate in the GWAS model will correct for confounding of statin and yield a change in association at rs7412. The response of the confounded signal was successfully diminished from *p* = 3.19 × 10^−7^ to *p* = 1.76 × 10^−5^, by accounting for statin using the scoring procedure presented here. This approach provides the ability for researchers to account for concomitant medications in complex trial designs where monotherapy treatment regimens are not available.

## Introduction

Patient-to-patient variability in responses to medicines is common, underscoring the need to develop more targeted therapeutic interventions, which is the principal aim for precision medicine initiatives (Collins and Varmus, 2015). However, conducting clinical trials to identify and support precision medicine interventions can be very costly and time consuming, and may be impractical if the response or disease is rare. An attractive alternative is to leverage biobanked samples from completed or ongoing clinical trials to conduct genome-wide association studies (GWAS) in order to identify genetic determinants of variability in drug response, potentially garnering additional value from the initial clinical trial investment.

Biobanks store and manage collections of human specimens, including but not limited to, human serum and plasma, solid tissues, blood, and bone marrow. As technology improves over time, research conducted using stored samples may facilitate medical breakthroughs. A 2012 survey of 456 US biobanks found that 59% of them had been established since 2001 and that they range in size from tens of specimens to over 50 million specimens (median of 8000) collected from as little as a few individuals to as many as 10 million individuals per biobank (Henderson et al., 2013). The large and growing number of available samples and the declining costs of genotyping provide attractive opportunities for retrospective genetic analyses (Jansen et al., 2005).

Biobanks collected during the course of clinical trials typically provide data on medication usage and compliance as well as relevant clinical outcomes for subjects from whom samples were banked, and thus provide a rich opportunity for investigating the genetics of drug response. However, as these trials were designed for testing only the efficacy and safety of drugs in a particular therapeutic setting and not for testing the genetic contribution to drug response, they also present significant challenges. Many clinical trials are designed to test efficacy of one or more treatment strategies involving combinations of drugs, and may not consist of individuals on a monotherapy treatment regimen. Thus, patients may be taking multiple medications, with potentially overlapping therapeutic targets, which can make it difficult to tease out the genetic determinants of the response to individual medications. Clinical trials typically use the Intention-to-Treat (ITT) approach to analysis, which results in ignoring issues of concomitant medications and medication compliance after initial subject randomization (Detry and Lewis, 2014). ITT can be a conservative drug efficacy testing approach as it mimics challenges associated with real-world clinical scenarios (Detry and Lewis, 2014), but for testing the association of genetic variants with variation in drug response, ITT poses a significant confounding risk.

In this study, we used frozen samples and data from the Action to Control Cardiovascular Risk in Diabetes (ACCORD) trial. The ACCORD study tested three treatment approaches, in a double two-by-two factorial design, to determine the best ways to decrease the high rate of major cardiovascular disease (CVD) events among individuals with type 2 diabetes (T2D), who are at especially high risk of having a CVD event, like a heart attack, stroke, or death. These three treatment approaches were: intensive lowering of blood sugar levels compared to standard blood sugar treatment; intensive lowering of blood pressure compared to standard blood pressure treatment; and treatment of blood lipids with two drugs—a fibrate plus a statin—compared to one drug, a statin alone (Buse, 2007). The ACCORD trial failed to demonstrate a reduction in adverse cardiovascular events with the intensive treatments, and the intensive glycemia arm was terminated early due to an increase in mortality (Gerstein et al., 2007). However, variability in response was observed in all treatment arms, and understanding the underlying genetic contribution to this variation in response may lead to more targeted and safer therapies.

ACCORD represents an especially challenging example of the difficulties encountered in conducting retrospective analyses of the genetics of drug response in a major clinical trial. The intensive glycemia and blood pressure lowering strategies involved treating to lower targets for glycated hemoglobin and systolic blood pressure than did the standard treatment strategies, the targets for which reflect those normally achieved in clinical practice. As such, neither strategy involved treating with predefined combinations of drugs, but rather, a variety of drugs could be used and could be added to existing therapy as needed at any time during the trial in an attempt to achieve treatment targets. Furthermore, individual drugs could be dropped from an individual subject's treatment plan at any time to deal with intolerable side effects, poor compliance, or in an attempt to find a more efficacious treatment combination for the individual subject. Thus, each subject had an individualized treatment trajectory, and despite the large size of the overall trial, it is essentially impossible to identify a large enough subset of subjects who initiate a particular medication (the primary medication) and maintain it long enough to measure a response, all on the background of an invariant set of concomitant medications. Instead, we have taken the approach of identifying the largest possible subsets of subjects starting and maintaining a given medication, and accounting for all other medications in our analyses. To do this, we needed a scoring procedure that was flexible enough to account for the different trajectories of each of the concomitant medications, i.e., whether the subject was already taking a particular concomitant medication before they start the primary medication or whether they start the concomitant medication at or after the time the primary medication is started, and whether they continue the concomitant medication with good compliance throughout the period in which the primary medication is being evaluated or whether they stop taking the concomitant medication at some point during that period. Depending upon the details of a concomitant medication's treatment trajectory, it may augment, diminish, or have no effect on treatment response to the primary medication, and each of these possibilities must be taken into account for each concomitant medication in the models used for genetic analysis.

We developed and applied our scoring procedure using several models of drug response in ACCORD. Two of these models involved the anti-hyperglycemic effects of metformin and of the thiazolidinediones (TZDs) rosiglitazone and pioglitazone. Metformin and TZDs are widely prescribed to lower blood glucose and increase insulin sensitivity in individuals with T2D and were prescribed to the majority of patients in ACCORD, often in combination with each other or with additional glucose-lowering drugs. In addition, we applied our scoring procedure to a model for response of blood lipids to fenofibrate. Our initial genetic association results, obtained without taking account of concomitant statin usage, yielded a significant association with the single-nucleotide polymorphism (SNP), rs7412, located in the *APOE* gene. However, previous studies have identified this SNP as being associated with LDL response to statin therapy (Postmus et al., 2014), and the majority of subjects in ACCORD were taking statins, suggesting that this result was largely due to statin usage, not fenofibrate. Controlling for statin using the scoring procedure described herein resulted in substantially diminishing the rs7412 association, further validating the scoring procedure.

## Methods

**Figure 1:**
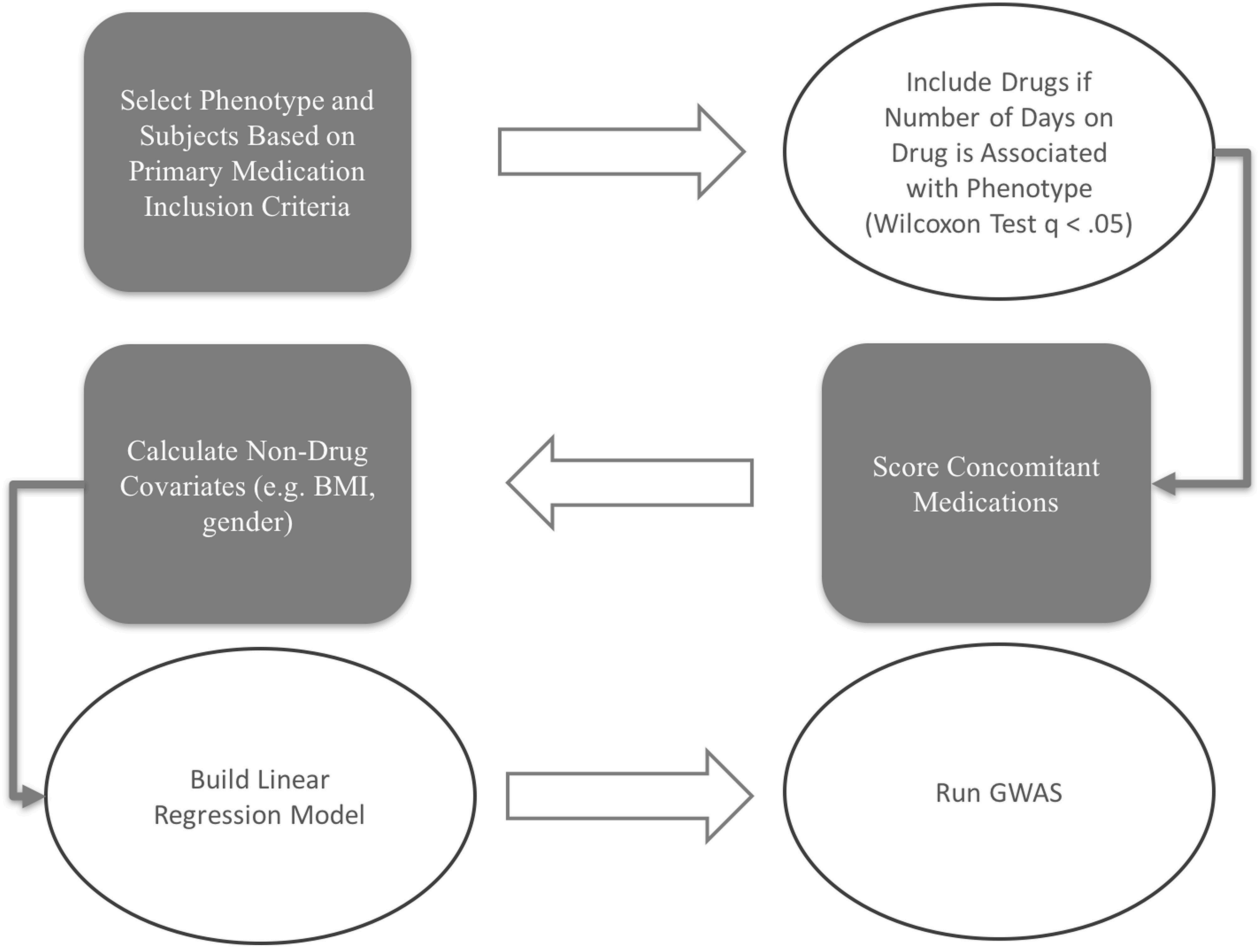
**Workflow Chart**.

The scoring and model building procedures workflow for this study is depicted in Figure 1.

### Accord trial data description

The ACCORD trial was a double 2 × 2 factorial design, consisting of 10,251 recruited subjects with T2D and either a history of CVD or at least two known risk factors for CVD, such as documented atherosclerosis, albuminuria, dyslipidemia, hypertension, smoking, or obesity (Buse, 2007). Subjects were randomized to either intensive or standard glycemia treatment strategies (targeting HbA1c<6.0 vs. HbA1c between 7.0 and 7.9). A subset of 4733 subjects were further randomized to intensive vs. standard blood pressure management (targeting systolic blood pressure of <120 mm Hg vs. < 140), and the remaining 5518 subjects were randomized to intensive vs. standard lipid management (fenofibrate vs. placebo, with all subjects on simvastatin).

Entry criteria for the lipid arm required an LDL of 60–180 mg per deciliter, an HDL < 55 mg per deciliter for women and black subjects or < 50 mg per deciliter for all other groups, and a TG < 750 mg per deciliter if not receiving lipid therapy or < 400 mg per deciliter if receiving lipid therapy (Ginsberg et al., 2010). The age range for subjects with a history of CVD was 40–79, and for those with no prior CVD history, 55–79. Body mass index (BMI) was limited to a maximum of 45, and serum creatinine to 1.5 mg per deciliter. Median length of follow-up was 4.7 years, and the primary outcomes were the first occurrence of nonfatal myocardial infarction or stroke, or death from cardiovascular causes.

### Phenotype and time frame selection

**Figure 2:**
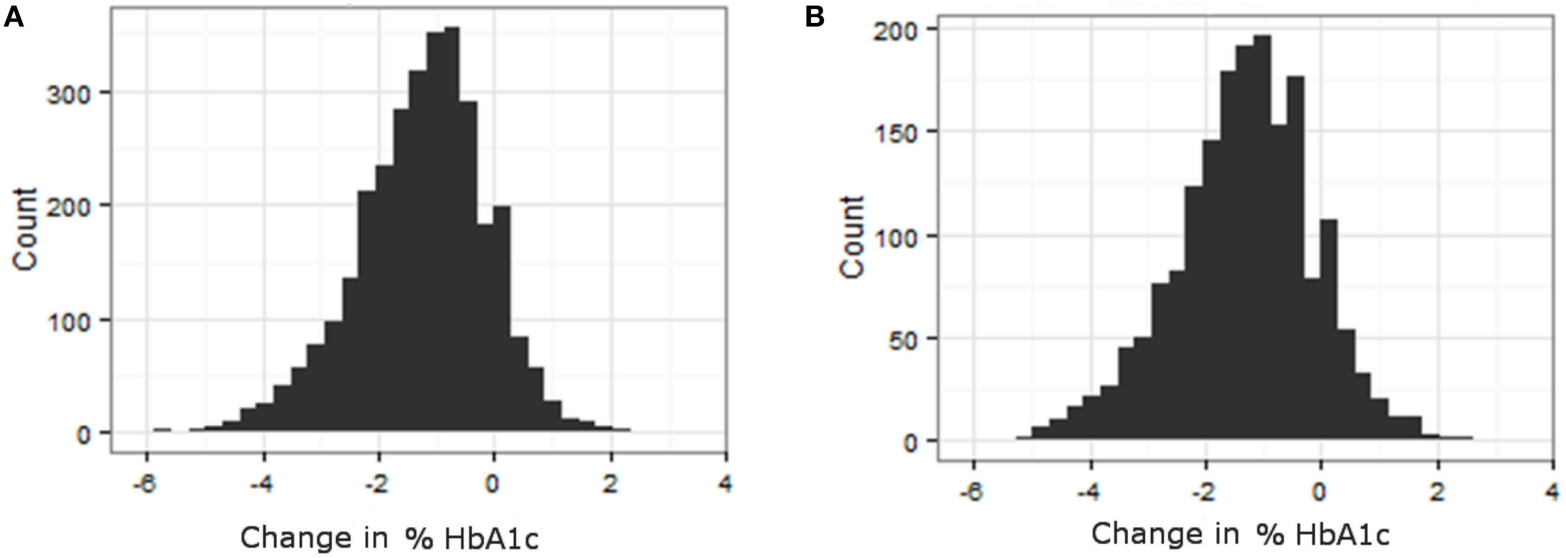
**Variability in response variable, change in %HbA1c, for all subjects that met the primary medication inclusion criteria as defined in the methods. (A)** Histogram of change in HbA1c for patients on TZD. **(B)** Histogram of change in HbA1c for patients on Metformin.

Phenotypes for analysis were based on medication efficacy. In both metformin and TZD analyses, the change in percentage of glycated hemoglobin (HbA1c) within a defined time period after starting the medication was used as the measure of medication effectiveness. Distributions of change in % HbA1c for metformin and TZD can be found in Figure 2. Initial phenotype values were defined as measured HbA1c at or within 30 days prior to starting the primary medication. Since HbA1c is a long term measure of glycemia, requiring red blood cell turnover to stabilize after a change in treatment, a minimum of 90 days of treatment was considered necessary to elicit the full effects of starting a new glycemia treatment on the measured phenotype. To minimize additional changes in treatment that were likely to occur in ACCORD during longer times on treatment, a maximum from initiation of treatment was set at 270 days. Therefore, final phenotype values were defined as the first measured HbA1c occurring between 90 and 270 days after medication initiation. Since the ACCORD trial spanned 8 years and subjects did not always commence a particular medication at the beginning of the trial, there is variability in when each subject's evaluation period occurred within the larger trial timeframe.

### Concomitant medication selection

A total of 93 concomitant medications or medication classes were identified as being used by subjects at some point during the ACCORD trial, either through subject self-report at baseline or annual physical exams or through reporting of study-supplied drugs on the study visit case report forms. These include all blood pressure, glycemia, and lipid lowering medications which were part of the ACCORD therapeutic interventions, as well as anticoagulants, anti-arrhythmics, NSAIDs, hormones, steroids, antidepressants, antipsychotics, over-the-counter medications and supplements, and other prescribed medications. To avoid having to score all 93 medications for inclusion in the drug-response models, we used the Wilcoxon Rank-Sum test to identify associations between a change in HbA1c and the number of days a patient was on each individual concomitant medication. Additionally, a false discovery rate controlling procedure was implemented to account for multiple comparisons. Significantly associated concomitant medications (*q* < 0.05) were then included in the model and scored according to the approach outlined herein.

Each Wilcoxon Rank-Sum test ignores other medications which may have impacted the association. Thus, we expect that this initial selection approach resulted in false positives, or drugs seemingly associated with the HbA1c change but that actually are not. However, the number of false negatives is likely limited since we expect the method to detect associations between time on medication and the phenotype. Thus, this is designed to be a conservative approach for initial variable selection to include any potentially confounding concomitant medications, which will be ultimately selected in the final model.

### Drug scoring

#### Cohort selection

In order to be included, all patients must have maintained full compliance, indicated as 80–100% compliance, in at least 80% of their recorded visits within the 90–270 day medication response time frame following the first record indicating use of the medication in the trial. Compliance was recorded in the ACCORD trial as a categorical variable. A value of 1 indicated 80–100% compliance, a value of 2 indicated 1–79% compliance, 3 indicated 0% compliance, and 4 indicated the patient took more than the prescribed dosage. For the purposes stated here, a value of 4 was considered to be full compliance (100%).

Missing values of compliance during the medication response measurement interval were imputed. The last observation carry forward (LOCF) procedure is typically used for missing-value imputation. LOCF carries the last non-missing observation forward to impute the missing value(s). However, in our study, if a compliancy record was missing, the next non-missing compliance value was backfilled for the missing-value imputation. This approach could be described as next observation carry backward (NOCB), and is preferable because patient compliance as assessed at each visit, is measured since the previous visit. If patient compliance in the interval after the missing record was maintained, then it was assumed the patient was likely compliant during the interval prior to the missing record, whereas, a prior record of compliance does not provide the same level of assurance regarding future compliance.

Additionally, any patient with any record of 0% compliance within the qualifying time frame was excluded to limit the possibility that poor drug response was due to poor compliance instead of genetic variability. A small number of patients were also excluded due to inconsistent records (*n* = 9 for both TZD and metformin analyses). For example, if a patient had records of taking the primary medications during their annual physical exam visits but no medication record could be found in their monthly visits, the patient was excluded from further analysis.

#### Concomitant medications

Concomitant medications selected using the Wilcoxon Rank Sum test (*q* < 0.05) were scored using the criteria in Table 1. In order to receive a score of 3 or 4 for concomitant medications, all patients must have maintained full compliance (100%) in at least 80% of their recorded visits within the designated time frame. If a compliance record was missing for a patient visit, the NOCB approach for missing-value imputation was applied, and the patient must not have had any 0% compliance record within the designated time frame.

**Table 1:** **Medication score descriptions**.

**Score**	**Score criteria**
0	The patient had no record of ever taking the medication of interest.
1	The patient was already on the medication at the start of the treatment-window and stopped taking the medication before the end of the primary medication treatment window.
2	The patient started the medication at or after the start of the treatment-window and stopped taking the medication before the end of the primary medication treatment window.
3	The patient started the medication at or after the start of the treatment-window and was compliant to the end of the primary medication treatment window.
4	The patient was already on the medication at the start of the treatment-window and was compliant to the end of the primary medication treatment window.

In determining the designated time frame, however, concomitant medications were required to use the exact time points as those established for the subject's primary medication. For qualifying concomitant medications, those with monthly records were scored using the logic found in Table 1. For concomitant medications with only a yearly drug record, patients not on the concomitant medication at the most recent record prior to starting the primary medication were assigned a 0; whereas, patients on the concomitant medication at the most recent record prior to starting the primary medication were assigned a 1. Some medications are represented in both sources. For example, the annual concomitant medication records include the TZD class of anti-hyperglycemia drugs, while individual TZDs are annotated in the medication logs filled out at the monthly visits. For drugs with both annual concomitant medication records and monthly visit medication logs, the annual records were used to determine whether a subject was already taking the medication at the start of the trial, and individual medications in the TZD class were combined to create a score for TZD medications.

The number of days subjects were on insulin was significantly associated with change in HbA1c. The glycemia management logs for the monthly visits in ACCORD require the clinician to record the average total dose of basal and bolus insulin (units per day) that the subject was taking since the prior visit. Since insulin usage was recorded as a continuous variable, it was not scored, but rather the change (in units) of total insulin per day was used in the variable selection.

### Non-drug covariate scoring

Medication scores were not the only covariates available for selection into the model. Other covariates including age, number of years with dyslipidemia, number of years with diabetes, smoking status, gender, clinical trial network, alcohol consumption, self-reported race, and education level were assessed at the start of the trial. Additional covariates, pre-treatment phenotype (HbA1c), BMI, average creatinine clearance, glomerular filtration rate, diastolic blood pressure, systolic blood pressure, waist size, serum creatinine, and fasting plasma glucose were recorded as the most recent measurement prior to starting the primary medication.

Population stratification, or systematic difference in allele frequencies between subpopulations due to ancestry differences, can provide false associations if not properly accounted for Price et al. (2010). Significant population stratification can oftentimes be observed, even within the same self-reported ethnicities or races. A method that can account for the differences in genetic ancestry between individuals without relying on self-reported measures is preferable. Principal components calculated from the genome-wide genotype data (see below) can be used to infer genetic ancestry and can therefore help avoid the negative consequences of population stratification (Price et al., 2010). Here, we allow the first 10 principal components (see Supplementary Material) to be selected into the model to avoid problems associated with population stratification, as described below.

#### Genotype data

The quality control and data processing steps are described in detail in Irvin et al. (2016). Briefly, genotypes were subjected to quality control to account for duplicate concordance, Mendelian segregation (in HapMap trios included on the genotyping plates), Hardy-Weinberg Equilibrium, and predicted gender. Cryptic relatedness was identified using KING (v1.4), and one member of each pair with a kinship coefficient > $\frac{1^{\frac{5}{2}}}{2}=$ 0.1768 was removed from the analysis data set (Manichaikul et al., 2010). Rare variant SNPs were excluded based on a minor allele frequency < 3%. Probes significantly deviating from HWE (χ^2^ > 19.51, *p* < 10^−5^) in at least two of the four main ethnic subgroups were excluded from the imputation process. The remaining untyped genotypes were prephased using SHAPEIT2 (v2.r778; Delaneau et al., 2012, 2013) and imputed using IMPUTE2 (Howie et al., 2009) to the 1000 genomes reference panel (Howie et al., 2012).

### Statistical model description

After all medications and other covariates were appropriately scored, a linear regression model was constructed using all covariates.

$$\hat{y}={\hat{\beta }}_{0}+{\hat{\beta }}_{1}x_{1}+{\hat{\beta }}_{2}x_{2}+\cdots +{\hat{\beta }}_{n}x_{n}$$

Two criteria were used for selecting observational units for the regression model: (1) any subject that met the primary medication inclusion criteria, and (2) any subject without missing covariate values. In cases where two covariates were collinear (|r| > 0.5), one of the covariates was dropped from the selection pool. Because concomitant medication scores are categorical, they are included in the model as dummy variables, meaning that each score is compared to the referent score of 0. Treatment arm indicators, principal components 1–3, to account for population stratification, and pre-treatment phenotype value were forced into the model.

Models using many covariates are prone to overfitting and use too many degrees of freedom, making replication of results difficult. Thus, a backward-selection approach was applied by comparing Bayesian information criterion (BIC) of the full model to the resulting BIC when removing one covariate. This comparison was repeated for each covariate, one at a time to determine which covariates had the least effect. Those with little effect were deemed insignificant and removed from the model. This is repeated for all covariates and the covariate that has the least effect on BIC is removed from selection in subsequent iterations. This is done until there is no longer a reduction in BIC when removing any of the remaining covariates.

### Proof-of-principle

As a proof-of-principle for the scoring procedures outlined above, a third medication, fenofibrate, was chosen for analysis, with simvastatin evaluated as a concomitant medication that was expected to be a confounder. Subjects in the lipid arm of ACCORD were randomized in a double-blind fashion to either fenofibrate or placebo. Patients receiving placebo were excluded from the analysis. Approximately 60% of subjects were taking a statin prior to entry into the trial, while all subjects in the lipid arm were put on simvastatin treatment at the trial baseline. Low-density lipoprotein (LDL) was the phenotype of interest because both fenofibrate and statins lower LDL. Final phenotype values were defined as the first measured LDL post-compliance for a time period of between 90 and 120 days. All other aspects scoring procedures were consistent with those described in Table 1.

Two linear models were run using the fenofibrate patients. Both models used backwards variable selection based on BIC. However, in the first model, statin scores were added to the list of forced covariates, while in the second, statin scores were removed from the covariate selection pool. After scoring and modeling was complete, genetic variants covering a five megabase region of chromosome 19 (chr19:42912079-47912079) and obtained as part of a GWAS (manuscript in preparation) were tested for association with response of LDL levels to fenofibrate. Previous studies identified a statin response with SNP rs7412, located in the APOE gene chr19:45412079. Here we hypothesize that including the statin score as a model covariate corrects for confounding of fenofibrate effect by statin and will yield an association change at rs7412. We used a statistical cutoff of *p* < 1 × 10^−6^, since this is routinely used as a threshold for suggestive significance in GWAS, and here we are focusing only on chr19, where the multiple testing burden is much lower, hence more conservative. SNPs do not satisfy the assumption of independence due to linkage disequilibrium, therefore standard multiple test correction methods (e.g., Bonferroni) are overly conservative.

We also tested a drug response of statin for rs7412, but since so many subjects were on statin prior to starting the trial, the sample size was small (*n* = 653). To determine if the results with and without statin correction were significantly different from one another, we performed 1000 bootstrap iterations to develop 95% confidence intervals around the β coefficient for rs7412. If the confidence intervals do not overlap then the difference is statistically significant. Additionally, we performed a Student's *t*-test between the two bootstrapped distributions to test if the means are significantly different, and a *p* < 0.05 was considered to be statistically significantly different.

## Results

The allocation of patients within the ACCORD trial arms has been previously reported (Buse, 2007). Baseline characteristics for patients included in the ACCORD trial are shown in Table 2. Females encompass approximately 40% of the trial and the majority of trial participants were Caucasian. 58.5% of subjects were currently or had previously been a smoker. The average diabetes duration was approximately 11 years and the average patient weight was 206 lb. Of the total 10,251 participants, 83% (8508) consented to provide biological specimens for future genetic analyses (Simons-Morton et al., 2014). After genotyping and quality control, genetic data for 7844 ACCORD participants were available for analysis. Medication compliance is a concern for any drug response study and non-compliance rates, defined here as any recorded compliance < 80% or greater than 100%, for TZD, metformin, and fenofibrate were 4.5, 4.2, and 13.6%, respectively. Additional details regarding the missing compliance and non-compliance rates are presented in Supplementary Table 1. Summary changes in %HbA1c for patients treated with TZDs or metformin are available in Supplementary Table 2.

**Table 2:** **ACCORD trial baseline characteristics**.

**Baseline characteristic**	**Overarching glycemia trial (*n* = 10, 251)**	**BP trial (*n* = 4733)**	**Lipid trial (*n* = 5518)**
Mean age (year)	62.77	62.73	62.79
Women (%)	38.55	47.71	30.70
Race/ethnicity			
White (%)	62.36	58.76	65.46
Non-white (%)	37.64	41.24	34.54
Mean duration of diabetes (yr)	10.80	10.99	10.63
Mean weight (lb)	206.16	202.82	209.02
Mean waist circumference (in)	42.02	41.61	42.37
Mean systolic BP (mm Hg)	136.15	139.00	133.70
Mean diastolic BP (mm Hg)	74.71	75.79	73.79
Mean HbA_1c_ (%)	8.28	8.31	8.26
Mean LDL-C, mg/dL	104.72	109.60	100.53

### Scoring results

#### TZDs

Of the original 10,251 ACCORD subjects, 2672 were excluded from the analysis due to one or more of the following reasons: patient medication information was not consistent across files (*n* = 9); the patient was taking a TZD, stopped for a period of time and then resumed the medication during the selected time frame (*n* = 439); the patient had at least one record of non-compliance (*n* = 379); the patient had an average compliance (after NOCB) of less than 80% within the selected time-frame (*n* = 809); the patient did not have a measured HbA1c value within the required time-frame (*n* = 1832).

Of the remaining 7579 subjects, 2411 were never prescribed a TZD during the trial, while 57 were not on a TZD for the minimum requirement of 90 days. The resulting 5111 subjects were compliant throughout the time-frame requirement. However, 2013 of these subjects were already taking a TZD at the beginning of the ACCORD trial. Thus, 3098 subjects met all inclusion criteria (i.e., started TZD during the trial, were compliant for the required period of time, and had valid starting and ending HbA1c measures) and were included in the subsequent analysis.

The concomitant medication distributions for eligible subjects taking TZD are shown in Supplementary Figure 2 and Table 3. Results from the initial concomitant medication screening as determined by the Wilcoxon Rank-Sum tests are also provided in Table 3. There were very few subjects taking “other diabetic medications,” while slightly more than half of the subjects were taking ACE inhibitors. Metformin, glimepiride, and statin all had scores of 4 for a large proportion of subjects, indicating subjects who were already taking these concomitant medications at the time they started a TZD.

**Table 3:** **Distributions of concomitant medications within TZD analysis**.

**Medication**	***p*-value^a^**	***q*-value^a^**	**Score^b^**					
			**0**	**1**	**2**	**3**	**4**	**Not scorable**
Sulfonylurea^c^	1.21E-10	1.13E-09	1011	344	37	166	1540	0
Meglitinide	2.20E-42	1.85E-40	3004	94	NA	NA	NA	0
Metformin	4.47E-12	4.70E-11	511	167	36	247	2137	0
Statin^d^	0.0042	0.0149	1441	56	4	98	1499	0
ACE inhibitors	0.0005	0.0023	1433	1651	NA	NA	NA	14
Other diabetic medications	0.0015	0.0064	3069	29	NA	NA	NA	0
Angiotensin II receptor blockers	0.0049	0.0169	2570	514	NA	NA	NA	14
Alpha-glucosidase inhibitors	3.50E-14	4.05E-13	3067	31	NA	NA	NA	0
Cholesterol absorption inhibitors	0.0115	0.0363	3012	5	NA	NA	NA	81
Lisinopril^e^	0.0054	0.0182	2607	81	24	117	269	0
Loop diuretics	0.0026	0.0101	2860	224	NA	NA	NA	14
Nitrates	0.0135	0.0421	2921	148	NA	NA	NA	29

a *Results from Wilcoxon Rank-Sum test for number of days on drug and change in %HbA1c across the whole trial*.

b *Cells with NA are from medications that only had yearly records and thus only have a 0 or 1 score*.

c *Individual medications from the sulfonylurea drug class were combined to create this score. The Wilcoxon Rank-Sum p- and q-values is for the sulfonylurea medication, glimepiride*.

d *Detailed statin records were only recorded for subjects in the lipid management arm of the trial, and was not recorded for the blood-pressure arm. However, statin were measured annually across all subjects, and is expected to capture this aspect of the trial design*.

e *Lisinopril was only recorded for subjects in the blood-pressure arm of the trial, and was not recorded for the lipid arm. However, Lisinopril is a member of the ACE Inhibitor class of drugs, which were measured annually across all subjects, and the ACE Inhibitor score is expected to capture this aspect of the trial design*.

Since the insulin scoring procedure yields a continuous variable, a histogram of the change in insulin usage in subjects starting TZDs is provided in Supplementary Figure 2. The values are tightly packed around 0 units with a standard deviation of 7.75 units indicating that few patients had large changes in insulin dosage. However, there are extreme values on either end creating long tails; the minimum insulin score is −57 units with a maximum of 110.5 units. The mean and median are 0.55 units and 0 units, respectively.

Of the 3098 subjects meeting the TZD inclusion criteria, only subjects that consented to genetic analysis were carried further, resulting in a total of 2431 subjects. The scored concomitant medications and other covariates were then tested for collinearity. Supplementary Table 3 shows variables with an |r| > 0.5 and outlines why Variable 1 was dropped. After collinear variables were removed from the pool of available covariates, a linear model was selected using a backwards selection method based on BIC. Table 4 shows the final model selected. Metformin was the only concomitant medication selected into the regression model.

**Table 4:** **Regression model selected for TZD analysis**.

**Variable**	**β**	**Std. Error**	***p*-value**
Intercept	−1.22	0.05	<2 × 10^−16^
Pre-treatment HbA1c	−0.67	0.02	<2 × 10^−16^
Principal component 1	−0.07	0.02	1.94 × 10^−4^
Principal component 2	−0.03	0.02	0.14
Principal component 3	−0.03	0.02	0.17
Baseline age	−0.07	0.02	1.98 × 10^−4^
Years diabetic	0.11	0.02	5.02 × 10^−10^
BMI	−0.11	0.02	4.12 × 10^−8^
Metformin score 1	0.03	0.10	0.80
Metformin score 2	−0.46	0.16	4.49 × 10^−3^
Metformin score 3	−0.47	0.08	1.79 × 10^−9^
Metformin score 4	−0.12	0.05	0.02

#### Metformin

Of the original 10,251 ACCORD subjects, 1843 were excluded from the analysis due to one or more of the following reasons: patient medication information was not consistent across files (*n* = 9); the patient was taking metformin, stopped for a period of time and then resumed the medication during the selected time-frame (*n* = 319); the patient had at least one record of non-compliance (*n* = 278); the patient had an average compliance (after NOCB) of less than 80% within the selected time frame (*n* = 919); the patient did not have an HbA1c value within the required time-frame (*n* = 802).

Of the remaining 8408 subjects, 772 were never prescribed metformin during the trial, while 74 were not on metformin for the minimum requirement of 90 days. The resulting 7562 subjects were compliant throughout the time frame requirement. However, 5740 of these patients were already taking metformin at the beginning of the ACCORD trial, so no pre-treatment HbA1c value was available. Thus, 1822 subjects met all inclusion criteria (i.e., started metformin during the trial, were compliant for the required period of time, and had valid starting and ending HbA1c measures) and were included in the subsequent analysis.

The concomitant medication distributions are shown in Supplementary Figure 1 and Table 5. The population for these distributions describes the 1822 patients who met the inclusion criteria for metformin. Results from the concomitant medication screening as determined by the Wilcoxon Rank-Sum test (*q* < 0.05) are also provided in Table 5. For subjects starting metformin, there were relatively few subjects taking meglitinide, TZDs, or “other diabetic medications,” while slightly less than half were taking ACE inhibitors. Glimepiride and statin have scores of 4 for a large proportion of subjects, indicating subjects who were already taking these medications at the time they started metformin.

**Table 5:** **Distributions of concomitant medications within metformin analysis**.

**Medication**	***p*-value^a^**	***q*-value^a^**	**Score^b^**					
			**0**	**1**	**2**	**3**	**4**	**Not scorable**
Sulfonylurea^c^	1.21E-10	1.13E-09	888	164	29	144	597	0
Meglitinide	2.20E-42	1.85E-40	1760	62	NA	NA	NA	0
TZDs^d^	1.97E-27	1.33E-25	1231	61	17	200	313	0
Statin^e^	0.0042	0.0149	918	22	3	78	801	0
ACE inhibitors	0.0005	0.0023	922	887	NA	NA	NA	13
Other diabetic medications	0.0015	0.0064	1782	40	NA	NA	NA	0
Angiotensin II receptor blockers	0.0049	0.0169	1517	292	NA	NA	NA	13
Alpha-glucosidase inhibitors	3.50E-14	4.05E-13	1812	10	NA	NA	NA	0
Cholesterol absorption inhibitors	0.0115	0.0363	1763	31	NA	NA	NA	28
Lisinopril^f^	0.0054	0.0182	1520	43	24	98	137	0
Loop diuretics	0.0026	0.0101	1644	165	NA	NA	NA	13
Nitrates	0.0135	0.0421	1696	98	NA	NA	NA	28

a *Results from Wilcoxon Rank-Sum test for number of days on drug and change in %HbA1c across the whole trial*.

b *Cells with NA are from medications that only had yearly records and thus only have a 0 or 1 score*.

c *Individual medications from the sulfonylurea drug class were combined to create this score. The Wilcoxon Rank-Sum p- and q-values is for the sulfonylurea medication, glimepiride*.

d *Individual medications from the TZD drug class were combined to create this score. The Wilcoxon Rank-Sum p- and q-values is for the TZD medication, rosiglitazone*.

e *Detailed statin records were only recorded for subjects in the lipid management arm of the trial, and was not recorded for the blood-pressure arm. However, statin were measured annually across all subjects, and is expected to capture this aspect of the trial design*.

f *Lisinopril was only recorded for subjects in the blood-pressure arm of the trial, and was not recorded for the lipid arm. However, Lisinopril is a member of the ACE Inhibitor class of drugs, which were measured annually across all subjects, and the ACE Inhibitor score is expected to capture this aspect of the trial design*.

A histogram of the change in insulin usage for individuals starting metformin is provided in Supplementary Figure 1. The distribution of values displays little deviation from 0 units with a standard deviation of 8.9 units, but there are extreme values on either end creating long tails. The minimum insulin score is −67.62 units with a maximum of 90 units. The mean and median are −0.34 units and 0 units, respectively.

Of the 1822 subjects meeting the metformin inclusion criteria, only subjects that consented to genetic analysis were carried further, resulting in a total of 1468 subjects. The scored concomitant medications and other covariates were then tested for collinearity. Supplementary Table 4 shows variables having an |r| > 0.5 and outlines why Variable 1 was dropped. After collinear variables were removed from the pool of available covariates, a linear model was selected using a backwards selection method based on BIC. Table 6 shows the final model selected. TZD was the only concomitant medication selected into the metformin regression model.

**Table 6:** **Regression model selected for metformin analysis**.

**Variable**	**β**	**Std. Error**	***p*-value**
Intercept	−1.39	0.03	<2 × 10^−16^
Pre-treatment HbA1c	−0.74	0.03	<2 × 10^−16^
Principal component 1	−0.11	0.02	6.67 × 10^−6^
Principal component 2	−0.07	0.02	8.46 × 10^−3^
Principal component 3	−0.04	0.02	0.07
Years diabetic	0.16	0.03	1.09 × 10^−10^
TZD score 1	0.49	0.14	6.21 × 10^−4^
TZD score 2	−0.52	0.32	0.10
TZD score 3	−0.43	0.08	9.33 × 10^−8^
TZD score 4	0.03	0.07	0.70
Glomerular filtration rate	−0.07	0.03	6.30 × 10^−3^

### Proof-of-principle results

The same scoring procedure described for the anti-hyperglycemia medications, above, was also applied to an analysis of fenofibrate drug response. Included subjects were participants in the fibrate arm of the Lipid subtrial of ACCORD. After selecting the fenofibrate cohort, and its corresponding secondary medications and all other covariates were scored, the variables were tested for collinearity. Supplementary Table 5 shows variables that have an |r| > 0.5 and outlines why Variable 1 was dropped. After collinear variables were removed from the pool of available covariates, a linear model was selected using a backwards selection method based on BIC. Table 7 shows the final model selected when statin score is a forced covariate and when the statin scores were removed from selection. The BIC for the model with statin was 1932, while the model without statin was 2113. This indicates that including statin obtained a better model fit, despite the incorporation of additional covariates.

**Table 7:** **Regression models selected for fibrate validation analysis**.

**Variable**	**With statin BIC = 1932.32**			**Without statin BIC = 2112.53**		
	**β**	**Std. Error**	***p*-value**	**β**	**Std. Error**	***p*-value**
Intercept	−0.026	0.016	0.103	−0.040	0.004	<2 × 10^−16^
Intensive glycemia arm	−0.015	0.006	0.015	−0.015	0.006	0.011
Principal component 1	0.002	0.003	0.606	2.25 × 10^−4^	0.003	0.933
Principal component 2	0.002	0.003	0.600	0.004	0.003	0.142
Principal component 3	0.006	0.003	0.040	0.008	0.003	0.005
Pre-treatment LDL score	−0.077	0.004	<2 × 10^−16^	−0.099	0.003	<2 × 10^−16^
Years diabetic	−0.010	0.003	1.18 × 10^−3^	−0.007	0.003	0.018
Years with dyslipidemia	0.009	0.003	5.17 × 10^−3^	0.011	0.003	1.92 × 10^−4^
Statin score 1	0.070	0.029	0.016			
Statin score 2	−0.026	0.038	0.497			
Statin score 3	−0.073	0.017	1.02 × 10^−5^			
Statin score 4	0.018	0.016	0.261			

**Figure 3:**
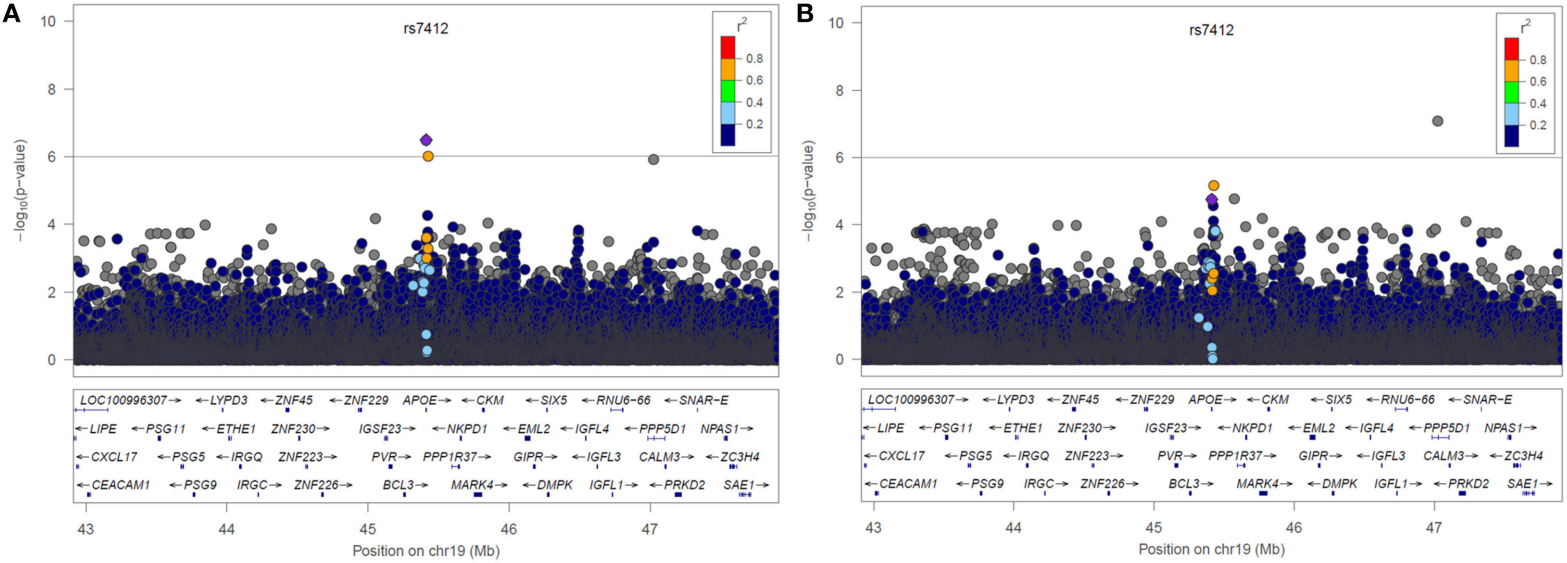
**LocusZoom plots of Chromosome 19 resulting from the GWAS (A) without statin included in the model and (B) with statin included in the model**.

LocusZoom plots of the selected region of chromosome 19 from the two resulting genetic association analyses can be found below in Figure 3. The lead SNP on the *APOE* gene (rs7412) is shown as a purple diamond. The yellow point above rs7412 is rs141622900 on the *APOC1* gene located directly to the right of *APOE* (~4 Kb). In the plot without statin as a covariate we see a peak indicating an association near the lead SNP on the *APOE* gene (*p* = 3.19 × 10^−7^, β = −0.0406, 95% CI[−0.0419, −0.0407]). In the plot when statin was included as a covariate, that association has been substantially reduced (*p* = 1.76 × 10^−5^, β = −0.0353, 95% CI[−0.0361, −0.0349]). There was a statistically significant difference between the two effect sizes (*p* < 0.001). We also tested for an association with statin drug response. Most subjects in ACCORD began statin treatment prior to starting the trial, preventing an appropriate pre-treatment measurement of LDL, subsequently 653 subjects meeting the inclusion criteria (analogous to fibrate). There was a significant association between SNP rs7412 and LDL response for subjects starting statin (*p* = 0.0016, β = −0.0357).

## Discussion

Here, we proposed a flexible scoring scheme to control which concomitant medications are selected and how they are accounted for in the analysis model for medication responses in a complex clinical trial. Although this method was incorporated into a linear regression model for the purpose of conducting GWAS with the ACCORD trial data, this scoring scheme is flexible and could be implemented into many statistical models.

We applied this scoring method to two commonly prescribed medications to treat T2D, TZDs, and metformin. In the TZD model, one concomitant medication, metformin, was selected for inclusion. Both of these drugs lower HbA1c, and are the two most commonly prescribed anti-diabetic medications in the ACCORD trial, so it is not surprising that metformin was incorporated into the TZD linear model as a concomitant medication.

In the metformin model, TZD was the only concomitant medication to be selected for inclusion. This is expected for the same reason that metformin scores were included in the TZD linear model. For the purposes of demonstrating the effect of the drug scoring, the trial arm variable was not included because it is specific to the ACCORD trial. Interestingly, in the TZD model, including the intensive glycemia arm indicator as a forced covariate removed the metformin score from the final model selection (not shown). Patients within the intensive glycemia arm of the trial were more likely to be taking additional medications than those in the other half of the trial. When the dummy variable for the intensive glycemia arm is added, metformin scores were no longer selected into the model. The inclusion of metformin once the intensive glycemia variable was excluded suggests that the variation contributed by metformin can be largely explained by the intensive glycemia arm variable.

A third medication, fenofibrate, was also tested along with appropriate concomitant medications and used as a validation of the approach described herein. Studies have shown that the use of statins lower LDL cholesterol, and although patient response to statins vary, part of this variability may be explained by genetics (Postmus et al., 2014). SNPs that have been shown to be associated with LDL response to statin treatment include rs2199936 (*ABCG2*), rs10455872 (*LPA*), rs7412 (*APOE*), rs445925 (*APOE*), and rs4420638 (*APOE*; Postmus et al., 2014). The rs7412 SNP located in the *APOE* gene was genotyped, along with surrounding SNPs, in ACCORD subjects as part of a GWAS (data not shown), allowing us to test the effect of incorporating statin as a covariate in an analysis of LDL response to fenofibrate. Thus, we hypothesized that we would find an *APOE* gene association using the model that does not account for the effects of statin, while the model with statin would not yield an association.

Two models for fenofibrate drug response were used as a proof-of-principle for the proposed drug scoring method, one with statin as a forced covariate and one without (Figure 3). In the model without adjustment for statin, an association of LDL levels with rs7412 in the *APOE* gene was observed (*p* = 3.19 × 10^−7^), which was significant based on our threshold of *p* < 1 × 10^−6^. The significance of this association was subsequently reduced to *p* = 1.76 × 10^5^ after incorporating the statin score into the model. We created bootstrapped confidence intervals around the β coefficients for the two models and the confidence intervals did not overlap, indicating the effect size was significantly reduced after correcting for statin (*p* < 2.2 × 10^−6^). Additionally, there was also a SNP (rs141622900) with nearly the same level of significance (*p* = 9.36 × 10^−7^) on the *APOC1* gene. The level of significance for rs141622900 was also diminished by adjusting for statin (*p* = 6.46 × 10^−6^). Previous studies also found that SNPs located on *APOC1* may contribute to the variation in LDL response with the use of statin (Barber et al., 2010). Although, most subjects started statin prior to entering the trial, 653 subjects were available and a significant association directly with statin treatment was also observed for rs7412 in this reduced sample size (*p* = 0.0016, β = −0.0357). Thus, these results demonstrate that the implemented scoring system works as expected.

In summary, the proposed scoring scheme worked well for the ACCORD trial data possessing a large number of study participants, detailed compliance, and concomitant medication data. The scoring scheme for when subjects start and stop medications in relation to the pre- and post-treatment values should be applicable to any drug response study design. Certain parameters (e.g., time–frame, compliance rate), could be readily modified to suite a specific study design that may be different than the ACCORD study presented here. Admittedly, for retrospective analyses of other biobanked clinical trial samples, the types and extent of data available will vary from trial to trial and thus, this scoring scheme may not be appropriate in all cases. In order to improve the sample size, the time-frame requirements could be relaxed. For example, lengthening the time-frame requirement would likely incorporate more patients, but would require a longer record of compliancy. Alternatively, tightening the time-frame would likely eliminate patients without a phenotype measurement, but would also reduce the length of time patients were required to be compliant. It may be informative to conduct future analysis using these scoring variations, test differences in selected models, and observe the quality of results.

Here we have proposed a flexible scoring procedure in order to incorporate both the presence and compliance of concomitant medications in linear regression models for the purpose of finding genetic contribution to drug-response variation. This procedure could be applied to many statistical models and has been shown to decrease the confounding effect of concomitant medications.

## Author contributions

DR, HG, and SM helped developed the methods used in the manuscript, performed analysis, and contributed to the manuscript writing. TH performed laboratory experiments. JB, AW, AR, and MW guided the research, helped develop methods and contributed to manuscript writing. The ACCORD/ACCORDion investigators conceived and conducted the ACCORD clinical trial. The complete list of Investigators can be found in Supplementary Material.

## Funding

Research was funded by NIH grants R01HL110380 and UL1TR001111.

### Conflict of interest statement

The authors declare that the research was conducted in the absence of any commercial or financial relationships that could be construed as a potential conflict of interest.
