# Supplementary material for: Incorporating Concomitant Medications into Genome-Wide Analyses for the Study of Complex Disease and Drug Response
Source: Front Genet. 2016 Aug 17;7:138. doi: 10.3389/fgene.2016.00138 (PMC5013254; doi:10.3389/fgene.2016.00138)
Supplement: Supplementary file 1 [file DataSheet1.docx]

Supplementary Material

Incorporating Concomitant Medications into Genome-Wide Analyses for the Study of Complex Disease and Drug Response

Hillary T. Graham^1, §^, Daniel M. Rotroff^1,2, §^, Skylar W. Marvel^2^, John B. Buse^3^, Tammy M. Havener^4^, Alyson G. Wilson^1^, Michael J. Wagner^4,*^, Alison A. Motsinger-Reif^1,2,*^,and the ACCORD/ACCORDion Investigators

^1^ Department of Statistics, North Carolina State University, Raleigh, NC, USA

^2^ Bioinformatics Research Center, North Carolina State University, Raleigh, NC, USA

^3^ Department of Medicine, University of North Carolina School of Medicine, Chapel Hill, NC

^4^ Center for Pharmacogenomics and Individualized Therapy, UNC Chapel Hill, Chapel Hill, NC, USA

**^§^** Equal contribution

*** Correspondence:**

Alison Motsinger-Reif, Ph.D.

NCSU Statistics Department

2311 Stinson Drive

Campus Box 7566

Raleigh, NC 27695-7566

919-515-3574

alison_motsinger@ncsu.edu

Michael J. Wagner, Ph.D.

1011 Genetic Medicine Building

120 Mason Farm Rd.

CB# 7361

Chapel Hill NC 27599-7361

919-966-9590

mjwagner@email.unc.edu

Supplementary Methods.

Population Stratification

Allele frequency differences due to population stratification can result in frequent false positive results or reduced power in genetic studies.(Tian et al., 2008) One well established way to compensate for these differences is to include principal components (PCs) as covariates when testing for associations. Principal component analysis reduces genotype data into a small number of orthogonal dimensions that describe as much variability as possible. The axes of variation can often be interpreted as describing differences in ethnic background or geographical location, although linear combinations of the top PCs may not be sufficient to capture this information.(Mathieson and McVean, 2012) EIGENSTRAT (v4.2)(Price et al., 2006) is used to compute the first ten PCs from a subset of genotyped variants. This subset consists of the variants that remain after filtering based on minor allele frequency (MAF) and linkage disequilibrium (LD) pruning using a variance inflation factor (VIF) threshold. The VIF threshold corresponds to 1*/*(1-R^2^) where R^2^ is the multiple correlation coefficient for a variant being regressed on all other variants simultaneously within a sliding window. PLINK is used to create a variant set with MAF *>* 0.01 and VIF *<* 1.5. The top ten PCs computed from the subset of variants are added to the candidate covariate list.

Supplementary Figures.


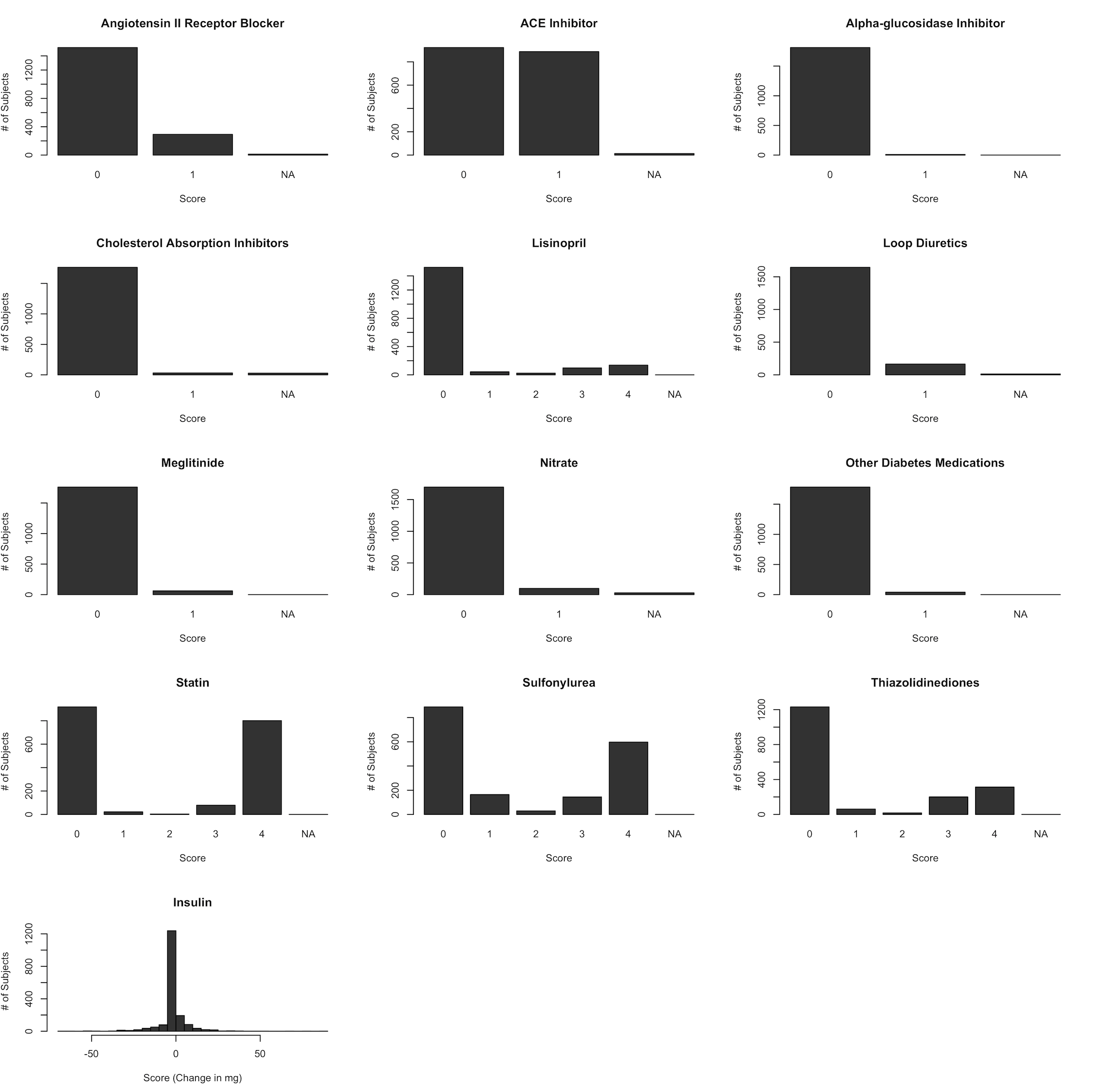


Supplementary Figure 1. Distributions of Concomitant Medications within Metformin Analysis. Very few patients were taking meglitinide, alpha-glucosidase inhibitors, or other diabetic medications during the time-frame for these subjects. Approximately half of subjects were taking ACE inhibitors. Sulfonylurea and statin have a large proportion of patients with a score of 4.


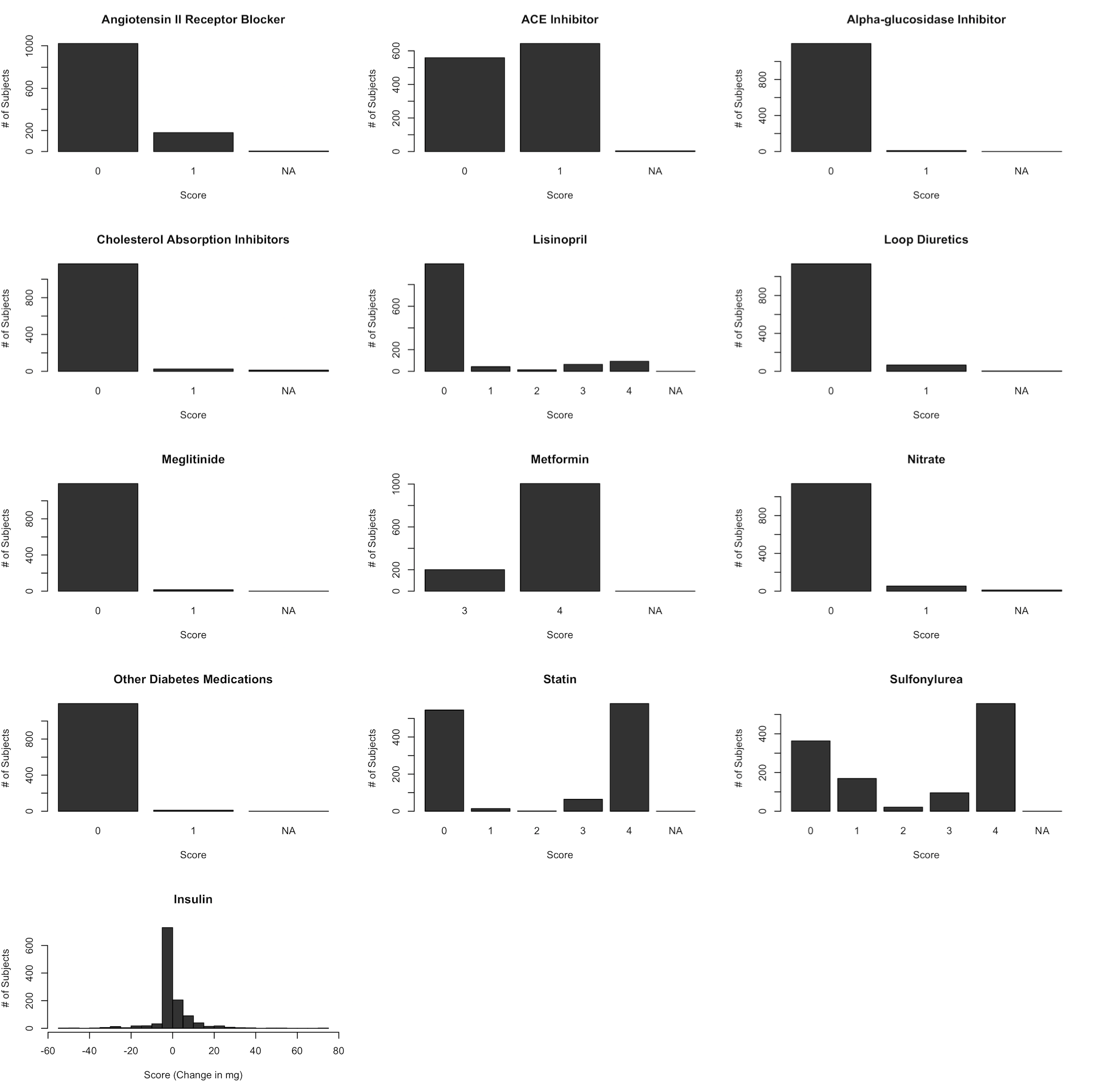


**Supplementary Figure 2.** Distributions of Concomitant Medications within TZD Analysis. Not many subjects were taking meglitinide, Alpha-glucosidase inhibitors, or other diabetic medications. More than half of patients were taking ACE inhibitors. Metformin, sulfonylurea, and statin have a large proportion of score 4 subjects.

## Supplementary Tables

**Supplementary Table 1.** Summary of Patient Compliance

| **Medication** | **Total Records** | **Missing Records** | **Missing Rate** | **Records Showing Compliance** | **Records Showing Non-Compliance** | **Non-Compliance Rate** |
| --- | --- | --- | --- | --- | --- | --- |
| TZDs | 194463 | 8119 | 0.042 | 178817 | 7527 | 0.042 |
| Metformin | 253244 | 4411 | 0.017 | 238191 | 10642 | 0.045 |
| Fenofibrate | 86243 | 9793 | 0.114 | 67301 | 9149 | 0.136 |

Supplementary Table 2 Summary Statistics for Response Variable, change in HbA1c (%)

| Primary Medication | Mean | Standard Deviation | Min | Max |
| --- | --- | --- | --- | --- |
| TZD | -1.24% | 1.13% | -5.7% | 3.1% |
| Metformin | -1.32% | 1.22% | -5.7% | 3.1% |

**Supplementary Table 3.** Correlation Table for Covariates within TZD Analysis

| **Variable 1 (dropped)** | **Variable 2**  **(kept)** | **Correlation** | **Reason** |
| --- | --- | --- | --- |
| Serum Creatinine | Glomerular Filtration Rate | 0.57 | Glomerular filtration rate incorporates serum creatinine measurements. |
| Waist Size (cm) | BMI | 0.68 | BMI incorporates the patient’s size in addition to other information. |
| Diastolic BP | Systolic BP | 0.56 | Systolic blood pressure has been shown to be a better predictor of risk of heart attack or stroke^1^. |
| Fasting Plasma Glucose | Pre-Treatment HbA1c | 0.54 | HbA1c is the outcome of interest. |

^1^(Mourad 2008)

Supplementary Table 4. Correlation Table for Covariates within Metformin Analysis

| Variable 1 (dropped) | Variable 2  (kept) | Correlation | Reason |
| --- | --- | --- | --- |
| Serum Creatinine | Glomerular Filtration Rate | -0.77 | Glomerular filtration rate incorporates serum creatinine measurements. |
| Waist Size (cm) | BMI | 0.64 | BMI incorporates the patient’s size in addition to other information. |
| Diastolic BP | Systolic BP | 0.56 | Systolic blood pressure tends to be a better predictor of risk of heart attack or stroke^1^. |
| Fasting Plasma Glucose | Pre-Treatment HbA1c | 0.51 | HbA1c is the outcome of interest. |

^1^(Mourad 2008)

Supplementary Table 5: Correlation Table for Covariates within Fibrate Validation Analysis

| **Variable 1 (dropped)** | **Variable 2**  **(kept)** | **Correlation** | **Reason** |
| --- | --- | --- | --- |
| Serum Creatinine | Glomerular Filtration Rate | -0.78 | Glomerular filtration rate incorporates serum creatinine measurements. |
| Waist Size (cm) | BMI | 0.64 | BMI incorporates the patient’s size in addition to other information. |
| Diastolic BP | Systolic BP | 0.55 | Systolic blood pressure has been shown to be a better predictor of risk of heart attack or stroke^1^. |
| Metformin | Biguanide | 0.75 | Metformin is in the biguanide class so should be incorporated in the biguanide score which is recorded annually and will preserve degrees of freedom in the model if selected. |

^1^(Mourad 2008)

References

Mathieson, I., and McVean, G. (2012). Differential confounding of rare and common variants in spatially structured populations. *Nat. Genet.* 44, 243–246.

Price, A. L., Patterson, N. J., Plenge, R. M., Weinblatt, M. E., Shadick, N. A., and Reich, D. (2006). Principal components analysis corrects for stratification in genome-wide association studies. *Nat. Genet.* 38, 904–909.

Tian, C., Gregersen, P. K., and Seldin, M. F. (2008). Accounting for ancestry: population substructure and genome-wide association studies. *Hum. Mol. Genet.* 17, R143–R150.
