# Supplementary material for: Incorporating Concomitant Medications into Genome-Wide Analyses for the Study of Complex Disease and Drug Response
Source: Front Genet. 2016 Aug 17;7:138. doi: 10.3389/fgene.2016.00138 (PMC5013254; doi:10.3389/fgene.2016.00138)
Supplement: Supplementary file 2 [file InvestigatorsList.docx]

**The Action to Control Cardiovascular Risk in Diabetes (ACCORD) and Follow-On (ACCORDION) Study Group**

***Steering Committee***: W.T. Friedewald (Chair), J.B. Buse (Vice Chair), A. Bertoni, J.T. Bigger, R.P. Byington, W.C. Cushman, F. Ismail-Beigi, S. Genuth, H. C. Gerstein, H. N. Ginsberg, D.C. Goff Jr, R.H. Grimm Jr, J.L. Probstfield, Y. Rosenberg, D.G. Simons-Morton.

***Canadian CCN****: Population Health Research Institute, Hamilton General Hospital, Canadian Diabetes Outcome Researchers (CANDOR Network), Hamilton, Ontario, Canada*: H.C. Gerstein, S. Yusuf, Z. Punthakee , R. Russo, S. Anand, K Chrysler

B. Cracknell, T. Cukierman-Yaffe, A. Gafni, G. Guyatt, S. Hall, J. Kaszyca, E. Lonn,

P. Mackie, V. Reiding, N. Shehadeh, B. Tadeson, K. Thompson, M. Vallis, V. Vasudeva, I. Wilderman, D. Wright.

***Canadian clinical sites***:

McMaster Medical Centre, Hamilton, Ontario, Canada: Z. Punthakee, A. Smith, I. Stanton, S. Capes, P. Manjoo, T. Valla, S. Danby, W. Harper, P. Harvey, V. Chaudhary, D. Hunt, Audrey Moroso, Rose Otto, Ally Prebtani.

*Six Nations Health Services, Ohsweken, Ontario, Canada*: Z. Punthakee, A. Davis, S. Capes, K.L. Hill, V. McCarthy.

*Diabetes, Hypertension and Cholesterol Centre, University of Calgary, Calgary, Alberta, Canada*: A.L. Edwards, R.J. Sigal, D.J. Mitchell, M.A. Clearwaters, C. Dielissen, M. Gillam, B. Hammond, H. Jensen, A. Kherani, D. Lau, V. Pringle, D. Rabi, R. Sigal, C. Smith, M. Walker, G. Williams.

*Memorial University of Newfoundland, St. John’s, Newfoundland, Canada*: C. Joyce, M. Parsons, B. Rowe, J. Burton, V. Chandurkar, S. CoadyMcDonald, D. Gibbons, C. Kovacs, B. Murphy, R. Smart, S. Varghese.

*University of Alberta, Edmonton, Alberta, Canada*: L. Mereu, E. Ryan, P. Senior, P. Kirkland, J. Abe, K. Dalton, W. Gendall, J. Germsheid, D. Hartmann, A. Jeffrys, U. Kumar, C. MacDonald, N. Makhani, S. Mawani, F. Morales, B. Paty, M. Pick, B. Schwanke, A. Stark, M. Tennant, S. Varma, D. Weiss-Aburto , P. WerbiskiWood, B. Woloschuk, W. Zimmerman.

*Centre de Recherche Clinique de Laval, Laval, Quebec, Canada*: A. Bélanger, S. Gauthier, G. Bahsali, C. Barbeau, E. Caponi, R. Duchesne, R. Dumas, P. Gauthier, J. Girouard, N. Kandalaft, M. Labbé, J. Palardy, M. Pilon, J. Raymond, A. Schiffrin.

*St. Joseph’s Health Care London, London, Ontario, Canada*: I. Hramiak, S. Tereschyn, M. Driscoll, M. Gehring, J. Gonder, C. Lincoln, W. McBeth, C. McDonald, T. McDonald, P. Pauli, T.Paul, S. Powers, N. Ronald, V. Trinh, L. Vancer, G. Walsh.

*Ottawa Hospital Research Institute, Division of Endocrinology and Metabolism, Ottawa, Ontario, Canada*: H. Lochnan, T.C. Ooi, J. Maranger, L. Bradley, R. Buhrmann, M. Cyr, C. Gilchrist, B. Hanlon, M. Harley, K. Jay, T. Leech, J. Malcolm, M. McLean, E. Parker, R. Sigal, K. Sullivan.

*Royal Victoria Hospital, Montreal, Quebec, Canada*: J.F. Yale, S.A. Segal, G. Al Ansari, N. Renouf, N. Allaire, M.A.M.A. Alawadhi, B. Belfer, D.W. Blank, F. Bouchard, S. BuoyPhang, J. Carter, L. Coppin, D. Dalpe, I. Delpech, P.M. Doran†, F. Emmian, S. Fortin, N. Garfield, M. Gosselin, S. Horan, M. Kalergis, S. Koutelias , C. Légaré, A. Lombardo, J.A. Morais, M. Quigley, N. Renouf, C. Riopel, S. Riopel, J.A. Rivera, G. Rochon, M. Roy, M. Salera, M.H. Sherman, M. Shingler, H.E. Staples, L. Ulyatt, Z. Yared.

*St. Michael’s Hospital, Toronto, Ontario, Canada*: L.A. Leiter, G. Booth, L. Sparrow, H. Choi, D.C. Bedard, A. Berger, L.A. Berndl, A. Cheng, V. Evalmplev, J. Goguen, A. Hanna, R.G. Josse, J.A. Kalas, S. Perry, M. Pike.

*Vancouver General Hospital, Vancouver, British Columbia, Canada*: T. Elliott, K. Dawson, J. Kong, D. Albiani, M. Inducil, R. Al Amoudi, T. Broughton, L. Hall, B. Harrison, N. Hirvi, R. Lee, A. Merkur, E. Norman, B. Paty, M. Potter, D. Stevenson, A. Vafadaran.

*Health Sciences Centre Diabetes Research Group, Winnipeg, Manitoba, Canada*:  V. Woo, L. Berard, T. Anderlic, K. Austman, A. Bernard, D. Catte, P. Darvill,D. Hak, K.Hutchison, L. Janzen, T. Klopak, C. Mandock, M. Mathen, S. Mawani, A. Mawani, L. Murphy†, G. Nyomba, B. Penner, S. Pockett, S. Russell, F. Stockl, J. Studney, R. Sukkau.

*Nova Scotia Health Authority, Queen Elizabeth II Health Sciences Centre, Halifax, Nova Scotia, Canada*: C. Abbott, E. Ur, M. Yuille, M. Archibald, D. Clayton, A. Cruess, N. Davis, H. Fong, S. Frizzell, B. Hanway, A. Hoskin-Mott, A. Imran, C. Ingraham, G. McCarthy, H. Murdock, T. Palmer, A.M. Patterson, T. Ransom, D. Shu, J. Tuttle.

***Western CCN***: University of Washington, Seattle, WA: J.L. Probstfield, C. Kingry, J. Dorje , Corson,  M.D. Sullivan, J. Johnson, A.S. Line, M.A., R. Knopp†, E. Lipkin, C. Griswold, K. Liebert, A. Brown, D. Juliano, E.M. Kurashige, S. Moberg, J. Leader.

***Western clinical sites***:

Northridge Hospital Medical Center, Cardiovascular Center, Northridge, CA: K. Ariani, D. Norman, K. Karunaratne, M. Azizad, C. Chow, H. Gutierrez, J. Partamian, J. Toven, J. Toven, J. Mular, S. Sanders.

*White Memorial Medical Center, Clinical Hypertension Services, Los Angeles, CA*: C. Gastelum, D.L. DeQuattro, L. Wang, L. Becerra, A., V. DeQuattro†, L.J. Haywood, V. Kamdar, Z. Song, Qi Cai, C. Pruitt.

*University of Washington Medical Center at Roosevelt, Family Medical Center, Seattle, WA*: R. Failor, C. M. Peterson, A. Ellsworth, N. Jackson, D. Britt, S. Dobie, I. Hirsch, D. Khakpour, R. Quaempts, W. Stoffel, W. Neighbor, K. Cappocia, V.Hawkins, L.Tapp.

*Idaho State University, Department of Family Medicine, Pocatello, ID*: R. Force, L. Chacon, E. Borzadek, J. Holmes, M. Macdonald, K. Pettingill, C. Liday, S. Koester, T. Pettinger, R. Solbrig, C. Waldron, W. Woodhouse, B. Hoover S. Lusk, T. Wilcox, D. Hachey.

*Naval Medical Center San Diego, Cardiology Division, San Diego, CA*: P.V. Pepper, S. Thompson, P.E. Linz,, C. Chase, D. Samuelson, P. Gutierrez, C. Gonzales, M. Engle, J. Coopersmith, S. Griffin, R. Lammers, J. Leon, D. Zirkle, S. Hollingsworth, J. Petrelli.

*Oregon Health & Science University, Section of Diabetes, Portland, OR*: M.C. Riddle, P.A. McDaniel, S.C. Gammell-Matthews, R. Swift, A.J. Ahmann, K.A. Hanavan , D.M. Karl, V. Burden, B. MacNeil, M. MacMurray, J. Weiss, C. Carlson, S.K. DesRochers, D. Negreanu, E.A. Stephens, D. Gale.

*Washington State University, Spokane, WA*: C. Wysham, J. Neumiller, D. Weeks, L. Weeks, M. Johnson, L. Kuntsmann, L. Maxwell, S. Yedinak, H. Pena, J. Kistler, J. White.

*Kaiser Endocrine Clinic, San Diego, CA*: J. Dudl, L. Lyons, P. Wu, B. House, M. Murray, R. Stevenson, A. Palma, S. Briere, T. Wilson, D. Becker, K. Harden, C. Hawley.

*Whittier Institute for Diabetes, Clinical Trials Department, La Jolla, CA*: G. Dailey, T. Vela, A. Gianella, M. Baron, M. Jacobson, E. Farro, A. Philis-Tsimikas, A. Banares, A. Bravo-Medina, J. Horne, E. Esquer, R. Morrissey.

***Minnesota-Iowa CCN***: Berman Center for Outcomes & Clinical Research, Minneapolis, MN: R.H. Grimm, Jr, B.R. Kirpach, M.M. Bartkoske, C.M. Boyce, N. Druckman, A.M. Gillett, J.A. Levin, G.J. Livingston, A.M. Murray, H. Wood, HealthPartners Research Foundation, Minneapolis, MN: K.L. Margolis.

***Minnesota-Iowa clinical sites***:

Hennepin ACCORD Clinic, Minneapolis, MN: S. Kempainen, M. Madden, M.Tariq Fareed, K. Hall, R. Moor, K.Wood.

*International Diabetes Center, Minneapolis, MN*: R. Bergenstal, R. Cuddihy, B. Davick, J. Hokanson, M. Johnson, M. Lausch, S. List, A. Monk, R. Robinson, K. Smith, D. Whipple, G. Damberg, R. Hahn, V. Koenig, M. Magadan, S. Sabin- Smith, P. Stewart, E. Strock, D. Peremislov, K. Gunyou, R. Passi . C. Ashanti, L. Thomas, D. Stoffels.

*University of Minnesota, Minneapolis, MN*: E.R. Seaquist, M.V. Mech, L.E. Benedict, D.J. Demmon, D. Kendall, A.F. Kumar, S.M. Martinson, S.A. Miller, C. Pease, J.P. Rao, J.B. Redmon, J.E. Swanson†, J.K. Wimmer, Y. Okorocha, M. Stiles, C. Kodl, C. Chadha.

*University of Minnesota, Phalen Village Clinic, St. Paul, MN*: KA. Peterson, L.A. Seaquist, C. Boese, M. Cruciani, E. Dodds, F. Parenteau Ek, J.L. Feldman, P. Fontaine, C.J. Lange, T.J. Mendenhall, A.M. Peterson, A. Rudelt T.M. Schrock, D.P. Spielman, S. Velasco, J.C. Weinhand.

*Riverside Health Partners Clinic, Department of Endocrinology, Minneapolis, MN*: J.M. Sperl-Hillen, P.J. O’Connor, M.E. Busch, A. Chung, B.K. Klein, N. Krugen, T. Bunkers-Lawson, H.L. Ekstrom, H.S. Gunderson, B.M. Johnson, J.H. MacIndoe, D.J. Prewedo, J.L. Rawl, C.M. Roethke, Mary Quinlan, C.R. Fox, B.M. Bate, Q.T. Cao, M.M. Ohnstad, P.J. Meyers, O.D. Fernandes.

*University of Iowa, Health Care Diabetes Clinical Research and Programs, Iowa City, IA*: W.I. Sivitz, S.M. Wayson, T.A. Lower, L. Larson, L.A. Ahrens, M. Bayless, S.E. Beck, J. Chahal, C. Chenard, G.C. Doelle, V.M. Guzman, U.M. Kabadi, K.A. Ochs, A. Rahhal, R.G. Spanheimer, L. Snetselaar, K. Smith, D. Wells.

***Ohio-Michigan CCN***: Case Western Reserve University, Division of Clinical and Molecular Endocrinology, Cleveland, OH: S. Genuth, F. Ismail-Beigi, M. Thibonnier, L. Vargo, C. Kelly, T. Bongorno, A. Dolish, L. Pavlik, M. Tiktin, S. Isteitieh A. Bartlett, T. Kulow.

***Ohio-Michigan clinical sites***:

University Hospitals of Cleveland, Division of Endocrinology, and University Hospitals Westlake Medical, Cleveland, OH: F. Ismail-Beigi, A. Krikorian, L. Moore, L. Richardson, E. Coles-Herman, K. Yee, J. Frankino, M. Jing, A. Sood, L. Hustak, M. Julius, L. Pavlik, T. Ross, L. Long, W. Schwing, M. Tiktin, M.K. Sullivan, L. Strauss, K. Behm, F. Eskandari, C. Hall, D. Hayes, K. Horowitz, S. Isteitieh, Z. Madhun,  E. Seeholzer, J. Shina, H. Taylor, A. Schnall, S. Huang, M. Heeg, J. Tang, J. Belkin, M.S. Lee, T. Joly, S. Solarz, T. Kulow, C. Patel, A. Pickett, A. Hagan.

*St. Vincent Charity Hospital, Lipid Research Center, Cleveland, OH*: L.S. Sadler, M. Griffith, A. Hornsby, K. Klyn, E. Ospelt, L. Long, M. DeSmit, P. McCann, N.P. Schmidt, C. Gottfried, T. Kulow, J. Zaletal, M.S. Kapadia, L. Smith.

*University Suburban Health Center, South Euclid, OH*: A.M. Schnall, L. Peysha, R. Ellert, J. Smith, J. Leksan, T. Sussman, S. Huang, M. Heeg, J. Tang, J. Belkin*, M.S. Lee, T. Joly.

*Cleveland Veterans Affairs (VA) Medical Center (VAMC), Department of Medicine, and Ravenna Community Based Outpatient Clinic, Cleveland, OH*: F. Ismail-Beigi, L. Hustak, M. Julius, W. Schwing, M. Tiktin, J. Anselmo, F. Eskandari, S. Daymeyer, C. Hall, D. Hayes, K. Horowitz, S. Isteitieh, C. Johnson, E. Kern, M.A. Richmond, L. Richardson, K. Roberts, J. Shina, A. Sood, P. Suhan, H. Taylor, S. Watts, J. Martin, L. Moor, B. Burtch, S. Ober, G.J. Strauss, A. Leone, J. Belkin, S. Huang, K. Frank, D. Stephens, M.S. Lee, T. Joly, S. Solarz, T. Kulow, C. Patel, A. Pickett, A. Werner.

*The Cleveland Clinic Foundation, Cleveland, OH*: B.J. Hoogwerf, J. Brakeman, M. Matzinger , J. Newsome, J. Becker, S. Bizjack, B. Clingman,  S. Curtas-Thomas, G. Depietro, R. Ellert, C. Horner, G. Bunae , A. Hamrahian, A. Hawkins, T. Head, S. Iannica, L. Jones, P. Kaiser,  R. McCoy, A. Mehta, L. Olansky, A. Orasko, S. Reddy, D. Ross, L. Shockley, E. Siraj, M. Williams, R. Zimmerman M.Hamaty.

*Your Diabetes Endocrine Nutrition Group, Mentor, OH*: D. Weiss, K.A. Fagan, T.M. Hanslik, J. Farrell, P. Brys, M. Oligny, K. Prokop, K. Lenardic, T. Karapanzcik,.

*Medical University of Ohio, Department of Medicine, Ruppert Health Center, Toledo, OH*: B. Akpunonu, R. Franco-Saenz†, J. Gilmore, M. Gilmore, L. Godfrey, P. Ross, B. Bauer, M. Chrisstie, A. Lopez, P. Mulrow, C. Peters, R. Pop-Busui, J. Roman, C. Smith, J. Bick, Z. Blust, P.T. Nelsen, D. Marcus.

*The Ohio State University Medical Center, Division of Endocrinology, Diabetes and Metabolism, Columbus, OH*: K. Osei, E.A. Dziengelewski, H. Breedlove, D. Boland, C. Casey Boyer, S. Cataland, P. A. Kearns, J.E. Irwin, D.P. Schuster, J.L. Varga-Spangler, T. Bowles, K. Weiland, K. Arnold; T. Evans, J. Bouttamy, A. Letson, E. Craig, F. Davidorf.

*University of Cincinnati/VA Medical Center, Research Service, Cincinnati, OH*: R.M. Cohen, K. Burton, J. Craig, B. Carter, J. Harrer, R. Hurd, D. Lopez-Stickney, C. Pritchard, A. Pfefferman, B.A. Ramlo-Halsted*, C. McCormick, C. Riley, M. Strominger**,** A. Knittel, G. Groff, C. Bailey, A. Howald , N. Anderson, J. Laver Bierschbach, M. Tyzinski*, B. Smith, S. Krug, V. Hershberger, R.K. Hutchins, L.A. Raymond, J. Bunke.

*Henry Ford Health System–New Center One, Detroit, MI*: A.Thomas, D.M. Kahkonen, T. Cushman, M. Roman, A.M. Stys, K. White, M. Austin, C. Chatterton, J.K. Francis, C. Jones, D. Kruger, A. McLellan, F. Whitehouse, E. Higgins, S. Levy, A. Schoenherr, P. Edwards.

*Grunberger Diabetes Institute, Bloomfield Hills, MI*: G. Grunberger, L.C. Aman, A.H. Bandagi, K.M. Russell, C. Tucker, Y. Abidova, A. Amirikia, M. Nardicchio, B. Billingsley, I. Hirjakova.

***Northeastern CCN***: Columbia University College of Physicians and Surgeons, New York, NY: J.T. Bigger, C.R. Lopez-Jimenez, R. Bornholdt, L. Busaca, H.N. Ginsberg, P. Gonzales, D. Gosh, P. Love†, A. Kosok, E. Robinson, R. Steinman, C. Watson, G. Reyes.

***Northeastern clinical sites***:

Jacobi Medical Center, Bronx, NY: U.K. Schubart, M. Mendoza, G. Goswami, A. Laufer, J. Russo, N. Vincenty.

*Albert Einstein General Clinical Research Center, Bronx, NY*: M.H. Alderman, L. Carroll, M.J. Sanguily†, J.U. Gorkin, A.C. Mayer, L. Ramos, V. Sessoms, A. Fritts Stewart.

*Cornell Internal Medicine Associates, New York, NY*: D. Brillon, J. Cordero, M.A. Richardson, E. Wei, F. Ganz, B.R. Meyer, J. Paley, S. Anderson, C. Charles, A. Dwoskin, R. Chiong, K. Hyams.

*The Diabetes Care and Information Center of New York, Flushing, NY*: D.L. Lorber, T. Arenstein, P. Depree, A.A. Elmorsy, J.M. Wendel, L.L. Zintl, P. August, M. Beck, M.D. Goldberg, M.J. Hofacker, M. Marotta-Kollarus, E.J.L. Ocampo, C.A. Resta, J.M. Tibaldi.

*The Cooper Health System, Cherry Hill, NJ*: A. Bastien, S. Grudzinski, P. Niblack, L. Abreu, T. Brobyn, K. Brown, M. Casale, D. Dougherty, G. Haddad, K. Heintz, M. Kelly, D. Linneman, C. Olivia, M.A. Salvador, P. Zee, D. Hyman.

*The Cooper Health System, Cherry Hill, NJ*: A. Bastien, S. Grudzinski, P. Niblack, L. Abreu, T. Brobyn, K. Brown, M. Casale, D. Dougherty, G. Haddad, K. Heintz, M. Kelly, D. Linneman, C. Olivia, M.A. Salvador, P. Zee, D. Hyman.

*Great Lakes Medical Clinic Research, Westfield, NY*: D.F. Brautigam, R. Fischer, J.M. Chiarot, D.M. Scharf, B. Nunn, J. Carlson, C. Flanders, M.R. Hagen, S. Newman, T.A. Gordon.

*Naomi Berrie Diabetes Center, New York, NY*: R. Goland, C.H. Tuck†, P. Kringas, J. Hey-Hadavi, J. Montes, J. Vargas-Jerez, J. Salas-Spiegel.

*Ambulatory Care Network at Columbia University, New York, NY*: A. Getaneh, J. Ramirez, E.F. Vasquez, G. Kranwinkel, S.A. Durant, Tang. Rocky.

*Irving Diabetes Research Unit, New York, NY*: D.S. Donovan, G. Febres, C. Hernandez, M.A. Jonaitis, L. Mesa. Tirado, Amilca.

*State University of New York Downstate Medical Center, Brooklyn, NY*: M.A. Banerji, M. Norton, P. Patel, V. Daly, S. Hirsch, C. Jazmin, R. Khillan, D. Mendonca, A. Relingado, E. Sandoval, M. Tiewala, Rambaud, Vahideh.

*Kings County, Brooklyn, NY*: M.A. Banerji, M. Norton, P. Patel, V. Daly, S. Hirsch, C. Jazmin, R. Khillan, D. Mendonca, A. Relingado, E. Sandoval, M. Tiewala, Rambaud, Vahideh.

*The Cooper Health System, Cherry Hill, NJ*: A. Bastien, S. Grudzinski, P. Niblack, L. Abreu, T. Brobyn, K. Brown, M. Casale, D. Dougherty, G. Haddad, K. Heintz, M. Kelly, D. Linneman, C. Olivia, M.A. Salvador, P. Zee, D. Hyman.

***Southeastern CCN***: Wake Forest University School of Medicine, Department of Public Health Sciences, Winston-Salem, NC: A. Bertoni, C.S. Blackwell, D.C. Goff, Jr, J.H. Summerson, L. Crago, R.L. Blaine, J.K. Kirk, R.L. Spach, J. Williamson, J. Calles, J. Katula, D.B. Wishnietsky.

***Southeastern clinical sites***:

Duke University Medical Center, Durham, NC: M.N. Feinglos, M.A. Furst, G. Gedon-Lipscomb, J. Jones, M.B. Mason, W.J. Bean, , J.B. Green, T. Parham, B.M. Satterwhite, C.R. Thacker.

*Constant Care, Inc., Valdosta, GA*: D. Padhiar, R. Noel, N. Padhiar, S. West, J. Braden, A Francis.

*Wake Forest University School of Medicine, Department of Geriatrics/Gerontology, Winston-Salem, NC*: W. Applegate, A.W. McFadden, H.H. Atkinson, M. Dibari, J. Allen, J. Stanfield, T. Delvalle-Fagan, L.J. Gordineer, L. Gordon, M. Gordon, S.L. Smith, H. Yates, K. Kennedy.

*Downtown Health Plaza, Winston-Salem, NC*: C.F. Pedley, G. Zurek, M. Baird, B. Dunn, W. Kinder, S. Mauney.

*University of North Carolina, Diabetes Care Center, Chapel Hill, NC*: J.B. Buse, K.D. Josey, D.C. Culmer, M.D. Duclos, R.E. Kirby, J.F. Largay, N.M. McDermott, A. Goley, S.S. Braithwaite, J.M. Dostou, E.A. Fasy, D.C. Kelly, C.E. Metz, J. Jeffries, D. Rubin, K. Vukojicik.

*Holston Medical Group, Kingsport, TN*: J.L. Miller, W. Besterman, S.M. Norton, J. Weatherly, S. Bishop, B. Cross, K. Nuss, M. Surgenor, D. Alley, A. Farmer, J. Foard, J. White, Y. Wood, B. Gross.

*Carolinas Medical Center Family Practice, Charlotte, NC*: M. Dulin, K. Preston, J. Konen, T. Barringer, S. Stanfield, K. Andrews, C. Hoffman, C. Morris, S. Norton, P. Tochiki, G. Reinblatt, P. Bruner.

*Robeson Health Care Corporation, Fairmont Clinic, Fairmont, NC*: R. Peace, D.O. Stuart, J. Strickland, L. Cummings, D. Craig, J. Stanfield, J. Morgan.

*Robeson Health Care Corporation, Julian T. Pierce Clinic, Pembroke, NC*: R. Peace, D.O. Stuart, J. Strickland, L. Cummings, D. Craig, J. Stanfield, G. Williams.

*Wake Forest University School of Medicine, Departments of Internal Medicine and Endocrinology, Winston-Salem, NC*: A. Bertoni, L. Menon, J. Calles, J.R. Crouse, S. Marion, D. Davis, B. Cabrera, T. Chandler, J. Ellis, E. Kouba, P. Riddle, E. Myers Mann.

*Tulane University Health Science Center, New Orleans, LA*: V. Fonseca, R.H. McDuffie, U. Japa, J. John-Kalaricka, N.O. Asafu-Adjaye, S.M. Leger, P. Reilly, G. Afner, F. Arrey, S. Asnani, E. Borshard, D. Boyd, A. Cemo, S. Chennur, P. Dupart, R. Garg, G.P. Girindra, B. Gouda, W. Itoua-N’Ganongo, I. Innocent-Ituah, C. Johnson, N. Kuhadiya, M. Kukreja, I. Mangan-Mbondi, S. Mason, C. McLain, J. Naylyanya, K. Nazereth, S. Nazereth, S. Singh, T. Thethi, K. Varnado, R. Williams.

*Kaiser Permanente, Clinic Atlanta Crescent Medical Center, Tucker, GA*: J.I. Barzilay, , K. Bader, D. Curry-Ball, S. Goodman, M. Curtis, M. Eley, C. Mayers, C. Ethridge.

***VA CCN***: Memphis VAMC, Memphis, TN: William C. Cushman, Cathy Thompson, Therese S. Geraci, Sandra M. Walsh, Linda G. Coley, Marshall B. Elam, Diane I. Pickering, C. Huff, Linda Shaw.

***VA clinical sites***:

Memphis VAMC, Hypertension/Lipid Research Clinic, Memphis, TN: M.B. Elam, C.W. Thompson, L. Lichtermann, S. Peeples, J. Turner-Bates, M. Heimberg, D. Childress, J. Turner, J. Jasper, J. Coley.

*Baltimore VAMC, Baltimore, MD*: B.P. Hamilton, J. Hamilton, G. Kuzbida, W. Hatten, Jr, A. Lancaster, J. Haywood, J. Luck, D. Bannerman-Wood.

*Carl T. Hayden VAMC, Phoenix, AZ*: J. Felicetta, M. Bourne- Collo, M.E. Svoboda, D. Clothier, M. Deitz†, C. Flaugher, P. Hayward, T. Scheibe, S. Velarde, S. Heritage, J.P. Nelson, D. Heritage, C. Martinez, S Aguayo, A Kuramoto.

*Atlanta VAMC Medical Service, Decatur, GA*: M.E. Sweeney, D. Harrelson, S. McConnell, F. Watson, R. Johnson, L. Whittington, M. Nanes, M. Salles, C Rice, C. Cowden, Maxine Maher.

*Ralph H. Johnson VAMC, Primary Care, Charleston, SC*: J. Basile, D.B. Ham, B. North-Lee, H.A. Baig, S.U. Rehman, J. Mixson, D Nelson.

*G. V. (Sonny) Montgomery VAMC, Research Department, Jackson, MS: K.A. Kirchner, L.A*. Hinton, L. Mack, C. Adair, B. James, A. Spencer, A. Jones.

VA NY Harbor Healthcare System, New York, NY: L. Katz, E.A. Richardson, A.G. Goldberg, A. Nieves, J.E. Russo, J. Adams, S.A. Sochalski, M.Coles, S. Anderson, M. Williams, D. Hoffman†, George Brock.

*Washington VAMC, Washington, DC*: V. Papademetriou, B. Gregory, R. Alignay, E. Nylen, B. Rajendran, R. Hodges†, S. Amodeo, A. Ross, A. Notargiacomo, M. Metcalfe, P. Narayan, D. Wojackovski.

*St. Louis VAMC, St. Louis, MO*: S. Giddings, E. Clark, A. Pittler, R. Davis, P. Harris, K. Waidmann, L. Conwill†, S. Felton, A. Chen, C. Rowe, K. Vargo.

*Central Arkansas Veterans Healthcare System,John L. McClellan Memorial Veterans Hospital, Little Rock, AR*: Michelle W. Krause, D.L. Simmons, J.J. Cooper, K. Dishongh, R. Bates, K. Bhaghayath, P. Choksi, S. Conley, S. Elbein, F. Faas, Z. Hamid, J. Johnson, P. Johnson, A. Mayo, M.S. Moriarty, G. Nair, D. Rani, N. Rasouli, S. Said, N. Rassouli, M. Rodriguez, K. Thomas, K. Watson, D. Williams, A. Makdissi.

***Coordinating Center:*** *Wake Forest University School of Medicine, Winston-Salem, NC:* R.P. Byington, W.T. Ambrosius, M.E. Miller, G. W. Evans, T. Craven, L.C. Lovato, D.E. Bonds, S. Wilmoth, L.T. Howard-Perdue, J. Hepler, J. Griffin, J. Beal, C. Stowe, A. Kimel, D. Cook, D. Dunbar, L. Sanders, J. Robertson, S. Rushing, J. Williamson, N. Woolard, R.T. Anderson, D. Beavers, J. Barnes, M. Barr, C. Bell, , S. Burton, H. Chen, S. Chen, C. Collins, B. Craven, P. Davis, P. Feeney, C. D. Furberg, C. M. Greven, L. Harvin, H. Hinkle, D. Hire, M. Howard, M. Hough, F. Hsu, W. Hwang, , I. Leng, D. Lefkowitz, A. Lopina, J. Lovato, T. Morgan, J. Pierce, D. Reboussin, L. Sims, M. Walkup, , K. Wilson.

***Drug Distribution Center***: VA Cooperative Studies Program Clinical Research Pharmacy Coordinating Center, Albuquerque, NM: D. Raisch, R. Ringer, M. Sather, B. DelCurto, D. Garnand.

***ECG Reading Center***: Wake Forest University School of Medicine, Winston-Salem, NC: R. Prineas, C. Campbell, Z. Zhang, L. Kesler, S. Hall, S. Hensley, Y. Li, E.Z. Soliman, D. Milne

***Central Chemistry Laboratory***: Northwest Lipid Research Laboratories, Seattle, WA: S. Marcovina, J. Harting, K. Gadbois, V. Gaur, G. Strylewicz, M. Ramirez, T. Speer.

***ACCORD-MIND MRI Reading Center***:  University of Pennsylvania, Philadelphia, PA: R.N. Bryan, C. Davatzkios, G. Moonis, L. Desiderio, S. D’Arcy.

***Fundus Photograph Reading Center***:  University of Wisconsin Medical School, Madison, WI:  M. Davis, R. Danis, S. Gangaputra, L. Hubbard, N. Robinson, J. Dingledine, D. Thayer, H. Wabers, M. Neider, B. Esser, T. Harding, R. Susman, C. Hurtenbach, V. Gama, M. Schiffman, S. Johnson, T. Graham.

***Project Office***:

National Heart, Lung, and Blood Institute (NHLBI), Bethesda, MD: D.G. Simons-Morton, L. Cooper, C. Nwachuku, Y. Rosenberg, M. Salive, P. Savage, J.L. Fleg, J.A. Cutler, N. Geller, D. Follmann, M. Proschan, C. Jennings, E. Schaeffer, P. Mills, J. Bittner, R. Kirby, P. Frommer†, L. Fine, A. Cristman, C, Schaupp, S. Banks.

National Institute of Diabetes and Digestive and Kidney Diseases (NIDDK), Bethesda, MD: J. Fradkin, S. Malozowski, C. Meyers, T. Hostetter, P. Savage.

National Institute on Aging (NIA), Bethesda, MD: L. Launer.

National Eye Institute (NEI), Bethesda, MD: E.Y. Chew.

Centers for Disease Control and Prevention (CDC), Atlanta, GA: A. Albright, K.M.V. Narayan, M. Engelgau, P. Zhang.
